# Assessing eating disorder symptoms in low and middle-income countries: a systematic review of psychometric studies of commonly used instruments

**DOI:** 10.1186/s40337-022-00649-z

**Published:** 2022-08-23

**Authors:** Camila Ospina Ayala, Camila Scarpatto, Claudia Milena Garizábalo-Davila, Paula Andrea Diaz Valencia, Tatiana Quarti Irigaray, Wilson Cañon-Montañez, Rita Mattiello

**Affiliations:** 1 Pontificia Universidade Católica Do Rio Grande Do Sul, Porto Alegre, Brazil; 2 Faculty of Nursing, Universidad de Antioquia, Medellín, Colombia; 3 Epidemiology Group, National College of Public Health, Universidad de Antioquia, Medellín, Colombia; 4 Universidade Federal Do Rio Grande Do Sul, Porto Alegre, Rio Grande do Sul Brazil

**Keywords:** Eating disorders, Validation, Questionnaires

## Abstract

**Background:**

Various well-validated interview and self-report instruments are available to assess eating disorder symptomatology. However, most psychometric studies have been conducted in high-income countries. The aim of the present study was to systematically review the available psychometric studies conducted in low- and middle-income countries on well-known measures for assessing eating disorder symptoms.

**Methods:**

Psychometric studies with the following instruments were included: the Eating Disorder Examination (EDE), the Eating Disorder Examination Questionnaire (EDE-Q), the Eating Disorder Inventory (EDI), the Eating Attitudes Test (EAT), and the Children’s Eating Attitudes Test (ChEAT). Searches were conducted on August 30, 2021, in the following databases: MEDLINE, EMBASE, LILACS, Web of Science, PsycINFO, and CABI. The methodological quality of the studies was assessed using the COnsensus-based Standards for the selection of health Measurement INstruments (COSMIN). The studies were considered to have conducted the minimum psychometric evaluation if they assessed at least the three types of validity (content, criteria, and construct) or diagnostic performance. The psychometric properties were also evaluated considering the cut-off points described in the literature for each of the analysis methods used to evaluate validity and reliability and two reviewers independently selected the studies and evaluated the quality criteria.

**Results:**

A total of 28 studies were included. The studies were conducted in 13 countries (10 middle income and 3 low income). The instruments that were most used in the studies were the EAT and EDE-Q. According to the overall COSMIN assessment, in most (57%) of the studies the psychometric properties assessed were not described. Forty-three percent of the studies conducted the minimum psychometric evaluation. However, according to the described cut-off points, the results for the psychometric properties assessed showed, in general, acceptable validity and reliability.

**Conclusion:**

The results of this review suggest a lack of studies with the recommended psychometric properties in low- and middle-income countries on these commonly used instruments. With the steady increase in the prevalence of eating disorders globally, psychometric investigations of instruments for measuring eating disorder symptoms in these countries should be encouraged to promote their early detection and treatment.

**Supplementary Information:**

The online version contains supplementary material available at 10.1186/s40337-022-00649-z.

## Plain English summary

The prevalence of eating disorders has increased worldwide. Various instruments are available to assess eating disorder symptomatology, but most psychometric studies have been conducted in high-income countries. The current study aimed to systematically review studies from low- and middle-income countries that have examined the psychometric properties of commonly used measures for assessing eating disorder symptoms. The findings of this study suggest a lack of research in low- and middle-income countries on the psychometric properties of commonly used eating disorder instruments. To promote the early detection and treatment of eating disorder symptoms, instruments with adequate psychometric properties must be available worldwide.

**Supplementary Information:**

The online version contains supplementary material available at 10.1186/s40337-022-00649-z.

## Background

Eating disorders (EDs) are psychiatric disorders characterized as disturbances in eating or eating-related behavior that lead to impaired consumption or absorption of food, which can compromise the physical and psychological health of the individuals affected [1–3]. EDs, particularly anorexia nervosa (AN), can in some cases lead to early mortality [4].

In a systematic review from 2019, the lifetime prevalence of EDs was estimated at 8.4% in women and 2.2% in men [5]. Although research has shown that the highest burden of EDs remains in high-income countries, prevalence studies have indicated that EDs have increased in other countries [6], specifically in East and South Asia [4, 7]. According to the results from the Global Burden of Disease Study 2017, low- and middle-income countries have shown an important increase in the age-standardized rate of ED prevalence, rising from 116.74 per 100,000 population in 1990 (95% UI: 92.25–548) to 156.96 in 2017 (95% UI: 123.42–194.26) [8]. However, the number of studies on the prevalence in low- and middle-income countries is limited [4]. Studies that help in identifying the local prevalence of eating disorders, such as in low- and middle-income countries, are very important; they can guide public health policies and help in implementing optimal and effective measures for the respective population to reduce the burden of this disease [8, 9].

The tools commonly used to evaluate EDs are the Eating Disorder Examination (EDE) [10], the Eating Disorder Examination Questionnaire (EDE-Q) [11], the Eating Disorder Inventory (EDI) [12], the Eating Attitudes Test (EAT) [13], the Children’s Eating Attitudes Test (ChEAT) [14], the Children’s Eating Disorder Examination (ChEDE) [15], and the Children’s Eating Disorder Examination Questionnaire (ChEDE-Q) [16]. Most studies that examine the psychometric properties of these instruments have been conducted in high-income countries and there is a scarcity of studies that show whether these instruments have been used in low- and middle-income countries. The use, in low- and middle-income countries, of instruments whose psychometric properties have not been evaluated prevents us from knowing whether the results of their measurements correspond to the actual state of the phenomenon being measured [17, 18] Therefore, the objective of the present study was to systematically review psychometric studies on instruments that assess ED symptomatology in low- and middle-income countries.

## Methods

### Protocol and registration

This systematic review was performed according to the Preferred Reporting Items for Systematic Reviews and Meta-Analyses (PRISMA) 2020 guidelines [19], and its protocol was registered in the International Prospective Register of Systematic Reviews (PROSPERO) under case number CRD42021219090 [20].

### Eligibility criteria

This systematic review included psychometric studies from low and middle-income countries. According to the World Bank in 2020, countries with low-middle-income economies are a diverse group in terms of size, population, and income level and are defined as having a gross domestic product per capita of between US$1,046 and US$4,095 [21]. This study covered the use of the EDE, EDE-Q, EDI, EAT, ChEAT, ChEDE, and ChEDE-Q instruments for assessing ED symptomatology. Review articles and duplicate publications were excluded. Articles were considered duplicates if they were in different databases and had the same Digital Object Identifier (DOI) or if they were from the same study group with the same inclusion period and individual study participant characteristics. In this case, the one with the largest sample size and the most recent publication date was considered.

### Information sources

A comprehensive search was conducted on August 30, 2021, in the following databases: MEDLINE (via PUBMED), EMBASE, Latin American & Caribbean Health Sciences Literature (LILACS via BIREME), Web of Science, PsycINFO (via APA PsycNET), and Commonwealth Agricultural Bureaux International (CABI). In addition, the references of the included studies and other systematic reviews were considered in the selection process.

### Search strategy

The MEDLINE search strategy was created and adapted for the other databases. There were no restrictions on language and year of publication. For the complete search strategy is presented in the attached online file 1 (see Additional file 1).

### Selection process

Two authors (COA and CMGD) independently scanned the abstract and title of each study from the search results. Next, all potentially relevant articles were read in full. In both phases, wherever there was a difference in opinion, a third author (RM), who did not initially evaluate the articles, reviewed them to reach a final decision.

### Data collection process and data items

Two authors (COA and CMGD) independently extracted the data. Any disagreements were presented to the third reviewer (RM) to establish a consensus. The following information was extracted: first author; year of publication; country; questionnaire language; eligibility criteria for participants; number of participants included (sample size); sex and age; instrument names; mode of administration of the questionnaire (self-reported or interview); number of items; domains assessed; psychometric properties; type of study. In the original protocol, we planned to contact the authors if we could not identify any of the information that was to be collected from the articles. However, all necessary information was presented in the included articles.

### Assessment of risk of bias in included studies

The methodological quality of individual studies was assessed according to the COnsensus-based Standards for the selection of health Measurement INstruments (COSMIN) [22]. The COSMIN checklist for assessing the methodological quality of individual studies contains ten sections, including translation process, content validity, hypotheses testing (or convergent and discriminant validity), structural validity (or construct validity), criterion validity (or diagnostic process), internal consistency, test–retest, measurement invariance, responsiveness, and, finally, the overall score for the COSMIN assessment. To classify the psychometric properties, a five-point scale with the following descriptors was used: “very good,” “adequate,” “doubtful,” “inadequate,” “not reported,” or “not applicable.” The overall score for the methodological quality of each measurement tool was determined considering the lowest classification for any one of the items evaluated. For example, for the structural validity criterion, if a confirmatory factor analysis (CFA) was conducted (implying a “very good” classification), but the sample size was < 5 times the number of items (meaning an “inadequate” classification), the general quality classification considered for that methodological property was “inadequate” [22].

We also considered that the studies conducted the minimum psychometric evaluation, according to international guidelines, if they assessed at least the three types of validity (content, criteria, and construct) or diagnostic performance was evaluated with a gold standard tool [23]. Psychometric studies that did not include a description of the translation process were considered as lacking content validity.

### Evaluation of psychometric properties evaluated in articles

The psychometric properties evaluated in the articles were assessed considering the cut-off points described in the literature for each of the analysis methods used to evaluate validity and reliability (Additional file 2).

The methodological quality of individual studies was visualized using the *robvis* web app, which depicts the plots obtained from these analyses [24]. The methodological quality of individual studies was assessed independently by two review authors (CA and CS). When there were differences in opinion, the third author (RM), who did not initially evaluate the articles, reviewed them to decide.

We assessed the performance of the original studies that developed and validated the questionnaires according to the COSMIN tool, minimum psychometric evaluation, and cut-off points.

We also described the methodological quality of individual studies according to the COSMIN tool of most validated questionaries’ and compared the COSMIN results for the validated questionnaires that underwent the translation process with those that did not undergo the translation process.

## Results

### Study selection

**Fig. 1:**
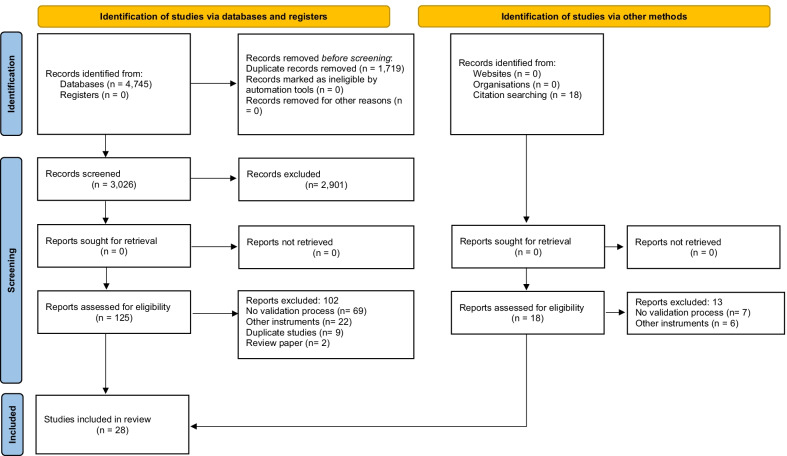
PRISMA 2020 flow diagram of study selection process

The search identified 4745 articles in the databases used, and a further 18 articles were identified in the gray literature. A total of 1719 duplicate articles were removed, leaving 3044 articles for the title and abstract evaluation. In this phase, 2901 articles were excluded. A further 1699 articles were excluded because they did not validate the instruments, 1026 used instruments other than the ones that are the focus of our study, 89 were studies conducted in populations from high-income countries, and 87 were review studies. Thus, 143 articles remained to be read in full. A further 115 were excluded because they did not meet the inclusion criteria: 76 studies did not validate the instruments, 28 used other instruments, nine were duplicates, and two were reviews. Thus, a total of 28 studies were included in this review. The systematic study selection is described in Fig. 1.

### Studies characteristics

The 28 studies included in this review comprising the research on psychometric properties were all cross-sectional studies published between 1989 [25] and 2021 [26–28]. One study validated the Eating Disorder Examination [29], ten validated the Eating Disorder Examination-Questionnaire [26–28, 30–36], four validated the Eating Disorder Inventory [37–40], 12 validated the Eating Attitudes Test [25, 41–51], and one validated the Children’s Eating Attitudes Test [52]. No study validated the Children’s Eating Disorder Examination [15] or the Children’s Eating Disorder Examination Questionnaire [16], and thus these two questionnaires are not further described here. The characteristics of the included studies are summarized in Table 1.

**Table 1:** Characteristics of included studies in this review

First author (Publication year)	Country	Questionnaire language	Eligibility criteria for participants	Sample size	Age, years, mean ± SD	Sex	Instrument	Mode of administration	Items	Domains	Evaluation of Psychometric properties according of Cut-off points
Tong [25]	China	Mandarin	Individuals with an EDs who came to Wuhan Hospital for psychotherapy or Wuhan Mental Health Center for treatment were included. Individuals with an EDs were diagnosed according to the DSM-IV criteria for AN or BN. The control group was undergraduate and graduate students from the China University of Geosciences in Wuhan without eating disorders	84	19.9 ± 3.2	Female/Male	Eating Disorder Examination	Interview	62	Restraint Eating Concern Shape Concern Weight Concern	Discriminant validity: AN BN Individuals without EDs Statistically significant difference Diagnostic performance: AUC not assessed Reliability Internal consistency: Cronbach's alpha coefficient: good Test–retest: Spearman coefficient: strong correlation
Penelo [31]	Mexico	Spanish	School children of both sexes (under 18 years old) of the Mexican population in Nayarit, in urban and rural areas. Participants were excluded if they did not answer the complete questionnaires	2928	15.1 ± 1.7	Female/Male	Eating Disorder Examination Questionnaire	Self-report	38	Restraint Eating Concern Shape Concern Weight Concern	Convergent validity: Questionnaire on Influences of Aesthetic Body Ideal: moderate correlation EDI-2: weak to strong correlation Children’s Eating Attitudes Test: moderate correlation Construct validity: Confirmatory factor analysis: χ2/df: adequate fit RMSEA: good fit CFI: mediocre adjustment Factor loadings: minimum level Measurement invariance: Model equal factor loading: χ2/df: adequate fit RMSEA: acceptable fit CFI: mediocre adjustment Model equal intercepts χ2/df: inadequate fit RMSEA: acceptable fit CFI: mediocre adjustment Reliability Internal consistency: Omega coefficient: good Test–retest: ICC: good Cohen’s Kappa coefficient: discrete
Becker [28]	Fiji	Fijian	The population of all available Fijian ethnic origins, aged 15–20, enrolled on Forms 3 to 6 in the 12 secondary schools registered in an administrative sector of the Fijian Ministry of Education in October 2006 were included	523	16.6 ± 1.0	Female	Eating Disorder Examination Questionnaire	Self-report	28	Restraint Eating Concern Shape Concern Weight Concern	Convergent validity: Fijian Body Shape Concern and Dissatisfaction Questionnaire: moderate correlation Questions on Tradition and Change: weak correlation Global School-Based Student Health Survey: weak to moderate correlation Construct validity Exploratory factor analysis: KMO and Bartlett’s test not evaluated Factor loadings: high level Reliability Internal consistency: Cronbach's alpha coefficient: moderate to good Test–retest: ICC: moderate Cohen’s kappa coefficient poor to substantial
Mahmoodi [30]	Iran	Persian	Female students from three medical universities of Tehran, Iran (including University of Social Welfare and Rehabilitation Sciences; Tehran University of Medical Sciences; and Islamic Azad University, Tehran Medical Branch) were included. Participants were excluded if they did not agree to participate or did not complete the instrument	516	23.7 ± 3.1	Female	Eating Disorder Examination Questionnaire	Self-report	28	Restraint Eating Concern Shape Concern Weight Concern	Convergent validity: Clinical Impairment Assessment: weak correlation Binge Eating Scale: moderate correlation Discriminant validity: Underweight students Overweight student Healthy weight student Statistically significant difference Reliability Internal consistency: Cronbach's alpha coefficient: excellent
Lewis-Smith [24]	India	English	Adolescents aged 11 to 15 years from four urban private secondary schools in Delhi, North-West India were included. Participants with > 10% of questionnaires items missing were excluded	1465	13.0 ± 0.8	Female/Male	Eating Disorder Examination Questionnaire	Self-report	28	Restraint Eating Concern Shape Concern Weight Concern	Convergent validity: Body Esteem Scale for Adolescents and Adults: rho: moderate correlation Construct validity: Exploratory factor analysis: Relative χ2: good fit RMSEA: acceptable to good fit CFI: mediocre adjustment to good fit TLI: mediocre adjustment SRMR: good fit Factor loadings: high level Confirmatory factor analysis: Relative χ2: inadequate fit RMSEA: good fit CFI: good fit TLI: mediocre adjustment SRMR: good fit Measurement invariance: CFI; RMSEA and SRMR not assessed Reliability Internal consistency: Cronbach's alpha coefficient: excellent Tests-retest: Cohen’s kappa coefficient: discrete to moderate
Unikel-Santoncini [26]	Mexico	Spanish	Women in psychiatric consultation at the Salvador Zubirán National Institute of Medical Sciences and Nutrition in Mexico City, diagnosed with AN or BN, were included. The control group was high school students without a diagnosis of eating disorders from a private school in Mexico City	495	19.3 ± 2.5	Female	Eating Disorder Examination Questionnaire	Self-report	28	Restraint Eating Concern Shape Concern Weight Concern	Construct validity: Confirmatory factor analysis: χ2/df: adequate fit RMSEA: acceptable fit CFI: good fit TLI: good fit SRMR: good fit Factor Loadings: high level Reliability Internal consistency: Cronbach's alpha coefficient: excellent
He [22]	China	Mandarin	Chinese undergraduate students under 25 years of age were included	1068	20.1 ± 1.0	Female/Male	Eating Disorder Examination Questionnaire–Short	Self-report	12	One-dimensional	Convergent validity: Eating Attitude Test‑26: r: moderate correlation Kessler Psychological Distress Scale: moderate correlation Construct validity: Confirmatory factor analysis: χ2/df: adequate fit CFI: mediocre adjustment TLI: mediocre adjustment RMSEA: mediocre adjustment Factor Loadings: high level Item response theory: Rasch modeling: Unidimensionality Variance explained: strong Unexplained variance: good Eigenvalue: acceptable Item calibration Inft: productive Outfit: productive Person separation index: good Person separation reliability: good DIF: intermediate to large Reliability Internal consistency: Cronbach's alpha coefficient: good Test–retest: ICC: good
Yucel [27]	Turkey	Turkish	Students belonging to primary and secondary schools representing low, medium, and high socioeconomic status in Istanbul were included	925	15.5 ± 1.8	Female/Male	Eating Disorder Examination Questionnaire	Self-report	28	Restraint Eating Concern Shape Concern Weight Concern	Convergent validity: Eating Attitudes Test: moderate correlation Turkish version of the general health questionnaire: moderate correlation Body image satisfaction questionnaire: null correlation Internal consistency: Cronbach's alpha coefficient: excellent Test–retest: Pearson correlation coefficient: very strong
Ramli [32]	Malaysia	Bahasa Malaysia	From four secondary schools, adolescents aged 12 to 17 were selected by stratified quota sampling to represent the Malaysian population with a ratio of race, gender, and academic performance were included. Specific ethnic groups were excluded	298	10 ± 8.2	Female/Male	Eating Disorder Examination Questionnaire	Self-report	36	Restraint Eating Concern Shape Concern Weight Concern	Construct validity: Confirmatory factor analysis: KMO: good Factor loading: minimum to high level Internal consistency: Cronbach's alpha coefficient: good
Mohd Taib [23]	Malaysia	Malay	University students from various disciplines in a public university in Klang Valley, Malaysia, were included. Participants were bilingual Malaysians (Malay—native language or English—instructional medium of the university)	595	21.9 ± 1.2	Female/Male	Eating Disorder Examination Questionnaire	Self-report	28	Restraint Eating Concern Shape Concern Weight Concern	Convergent validity: Eating Attitudes Test: moderate correlation World Health Organization Quality of Life Brief: weak correlation Construct validity: Exploratory factor analysis: KMO and Bartlett’s test not evaluate Factor loadings: minimum to high level Internal consistency: Cronbach's alpha coefficient: excellent Test–retest: Pearson correlation coefficient: strong
Compte [29]	Argentina	Spanish	Men living in communities in Argentina from four groups of college students, weightlifters, cross-fit gymnasiums, and rugby players were included	986	26.5 ± 6.7	Male	Eating Disorder Examination Questionnaire	Self-report	28	Restraint Eating Concern Shape Concern Weight Concern	Construct validity: Confirmatory factor analysis: χ2/df: adequate fit CFI: good fit TLI: mediocre adjustment to acceptable fit SRMSR: good fit RMSEA: acceptable to marginal fit Measurement invariance: Configural vs Metric: ΔCFI: non invariance ΔRMSEA: non invariance ΔSRMR: strong invariance Metric vs Scalar: ΔCFI: non invariance ΔRMSEA: non invariance ΔSRMR: strong invariance Internal consistency: Omega coefficients: good to excellent
Unikel-Santoncini [33]	Mexico	Spanish	Women diagnosed with an EDs according to the DSM-IV criteria through a clinical interview were included	523	19.9 ± 3.9	Female	Eating Disorder Inventory	Self-report	64	Drive for Thinness Bulimia Body Dissatisfaction Perfectionism Ineffectiveness Interpersonal Distrust Interoceptive Awareness Maturity Fears	Convergent validity: Symptom check list: moderate to strong correlation Coopersmith Self Esteem Inventory: null correlation Construct validity: Exploratory factor analysis: KMO and Bartlett’s test not evaluated Factor Loadings: high level Internal consistency: Cronbach's alpha coefficient: excellent
García-García [34]	Mexico	Spanish	Women diagnosed with an EDs according to the criteria of the DSM-IV, through a clinical interview were included	47	18.4 ± 3.6	Female	Eating Disorder Inventory—2	Self-report	91	Drive for Thinness Bulimia Body Dissatisfaction Perfectionism Ineffectiveness Interpersonal Distrust Interoceptive Awareness Maturity Fears Asceticism Impulse Regulation Social Insecurity	Diagnostic performance: AUC not assessed Internal consistency: Cronbach's alpha coefficient: good
Dadgostar [36]	Iran	Persian	Participants over 18 years of age from Iranian universities were included. Participants with questionnaires with more than 20% missing data were excluded from the analyses	452	22.3 ± 4.0	Female/Male	Eating Disorder Inventory—3	Interview	91	Drive for Thinness Bulimia Body Dissatisfaction Low Self-esteem Emotional Dysregulation Perfectionism Asceticism Interoceptive Deficit Maturity Fear Interpersonal insecurity Personal Alienation Interpersonal Alienation	Content validity: Validity index for clarity: acceptable standard Validity index for relevancy: acceptable standard Comprehensiveness of the survey: acceptable Internal consistency: Cronbach's alpha coefficients: questionable to good Test–retest: ICC: moderate
Rutsztein [35]	Argentina	Spanish	Women aged between 13 and 19 years old, from middle school in the City of Buenos Aires or Greater Buenos Aires, who agreed to participate in the study, and presented the informed consent signed by someone parental were included. Students who presented severe communication difficulties and questionnaire understanding were excluded	725	15.1 ± 1.3	Female	Eating Disorder Inventory—3	Self-reported	91	Drive for Thinness Bulimia Body Dissatisfaction Low Self-esteem Emotional Dysregulation Perfectionism Asceticism Interoceptive Deficit Maturity Fear Interpersonal insecurity Personal Alienation Interpersonal Alienation	Construct validity: Exploratory factor analysis: Eating Disorder Risk Scale: Bartlett's test: statistically significant KMO: very good Scales of psychological characteristics: Bartlett's test: statistically significant KMO: very good Internal consistency: Cronbach's alpha coefficient: questionable to excellent
Savaşır [21]	Turkey	Turkish	Women from the province of Ankara aged between 11 to 70 years who attended secondary schools, nursing colleges, medical schools, conservatories, dancers, and different socioeconomic levels were included. Elementary school students were excluded	745	23 ± 5.4	Female	Eating Attitudes Test-40	Self-reported	40	Dieting Bulimia and food preoccupation Oral control	Construct validity: Exploratory factor analysis: KMO and Bartlett’s test not evaluated Factor loadings: minimum to high level Internal consistency: Cronbach's alpha coefficient: acceptable
Nasser [37]	Egypt	Arabic	Girls aged between 15–16 years old from the first year EL-Nile Secondary is a State School were included	351	15.7 ± 7.7	Female	Eating Attitudes Test-40	Self-reported	40	Dieting Bulimia and food preoccupation Oral control	Construct Validity: Confirmatory Factor Analysis: Factor loadings: middle to high level Reliability: Internal consistency: Cronbach's alpha coefficient: unacceptable to good
Alvarez-Rayón [40]	Mexico	Spanish	Female participants aged between 15 and 29 years from outpatients with eating disorders treated at the Eating Disorders Clinic at the National Institute of Psychiatry in Mexico City seen in consultation and college or undergraduate students at the National Autonomous University of Mexico were included	556	19.3 ± 3.7	Female	Eating Attitudes Test-40	Self-reported	40	Dietary Restraint Bulimia Drive Thinness Food Preoccupation Perceived Social Pressure	Discriminant validity: Individuals with EDs Control Group Statistically significant difference Construct validity: Exploratory factor analysis: KMO and Bartlett’s test not evaluated Factor Loadings: middle to high level Diagnostic performance: AUC not assessed Reliability Internal consistency: Cronbach's alpha coefficient: excellent
Nunes [41]	Brazil	Portuguese	Women aged between 12 to 29 years from the city of Porto Alegre, Southern Brazil were included	163	24.2 ± 3.9	Female	Eating Attitudes Test-26	Self-reported	26	Dieting Bulimia and Food Preoccupation Oral Control	Diagnostic performance: AUC not assessed Reliability Internal consistency: Cronbach's alpha coefficient: acceptable
[42]	Brazil	Portuguese	Male adolescents aged between 10 to 19 years enrolled in private or public schools in Juiz de Fora, Brazil were included. Participants who did not answer the questionnaires in full or because they did not participate in anthropometric measurements were excluded	357	14.2 ± 2.2	Male	Eating Attitudes Test-26	Self-reported	26	Dieting Bulimia and Food Preoccupation Oral Control	Convergent Validation: Body Shape Questionnaire: fair correlation Discriminant validity: Low weight Normal weight Overweight Obese Statistically significant difference Construct validity: Exploratory factor analysis: Bartlett's Test: statistically significant KMO: very good Factor Loadings: minimum to high level Internal consistency: Cronbach's alpha coefficient: good Test–retest: ICC: excellent
Kang [44]	China	Mandarin	A clinical group of women aged between 13 to 29 years old who were born and lived in Mainland China and who met the diagnostic criteria for EDs were included. The control group was healthy women aged between 13 to 29 years old from schools and universities	802	19.6 ± 4.5	Female	Eating Attitudes Test-26	Self-reported	26	Dieting Bulimia and Food Preoccupation Oral Control	Convergent validity: Eating Disorder Inventory-I: moderate to strong correlation Diagnostic performance: AUC: high accuracy Internal consistency: Cronbach's alpha coefficient: good to excellent Test–retest: ICC: good
Constaín [45]	Colombia	Spanish	Women aged between 15 to 25 years old with criteria of AN and BN from the city of Medellín who attended a psychiatric consultation at a community care level were included. The control group was students from a private university in Medellín. Participants suffering from stupors, depression, catatonia, schizophrenia, neoplasms, HIV infection, malabsorption syndrome, untreated diabetes mellitus, uncorrected hypo, and hyperthyroidism or any other severe medical illness that was related to malnutrition and hypometabolism were excluded	136	20.6 ± 1.9	Female	Eating Attitudes Test-26	Self-reported	26	Dieting Bulimia and Food Preoccupation Oral Control	Construct validity: Exploratory factor analysis: Bartlett's test: Statistically significant KMO: very good Factorial loadings: high level Diagnostic performance: AUC: excellent accuracy Internal consistency: Cronbach's alpha coefficient: excellent
Constaín [46]	Colombia	Spanish	Man ≥ 14 years old with criteria of AN, BN and EDs not otherwise specified from the city of Medellín who attended a psychiatric consultation at the community care level were included. Control group was students from a private university in Medellín. Participants suffering from stupors depression, catatonia, schizophrenia, neoplasms, HIV infection, malabsorption syndrome, hypo and hyperthyroidism or uncontrolled diabetes mellitus, and any other pathology related to malnutrition and hypometabolism were excluded	114	21.8 ± 5.7	Male	Eating Attitudes Test-26	Self-reported	26	Dieting Bulimia and Food Preoccupation Oral Control	Construct validity: Exploratory factor analysis: Bartlett's test: Statistically significant KMO: average Factor loadings: middle to high level Diagnostic performance: AUC: excellent accuracy Internal consistency: Cronbach's alpha coefficient: good
Erguney-Okumus [47]	Turkey	Turkish	Male and female university students from the six different cities of Turkey were included	1500	20.6 ± 3.0	Female/Male	Eating Attitudes Test-26	Self-reported	26	Dieting Bulimia and Food Preoccupation Oral Control	Convergent validity: Eating Attitude Test-40: moderate correlation Brief Symptom Inventory: weak correlation Eating Disorder Rating Scale: moderate correlation Construct validity: Exploratory factor analysis: Bartlett's test: statistically significant KMO: good Factor loadings: minimum to high level Confirmatory factor analysis: χ2/df: adequate fit CFI: mediocre adjustment GFI: poor fit SRMR: good fit RMSEA: acceptable fit Internal consistency: Cronbach's alpha coefficient: questionable to good Test–retest: Pearson correlation coefficient: strong
Kaewporndawan [43]	Thailand	Thai	Individuals with EDs according to the DSM-IV-TR medical diagnostic, participants with other psychiatric disorders, and normal population were included	70	23.2 ± 5.5	Female	Eating Attitudes Test-26	Self-reported	26	Dieting Bulimia and Food Preoccupation Oral Control	Content validity: Item total correlation coefficient: excellent Discriminant validity: Individuals with eating disorders Control group statistically significant difference Diagnostic performance: AUC: excellent accuracy
Haddad [38]	Lebanon	Arabic	Participants above 18 years of age were randomly selected from each village were eligible to participate. Participants were excluded if they refused to fill out the questionnaire and those suffering from cognitive impairment reported by a family member	811	27.5 ± 11.7	Female/Male	Eating Attitudes Test-26	Interview	26	Dieting Bulimia and Food Preoccupation Oral Control	Construct validity: Exploratory factor analysis: Bartlett's test: statistically significant KMO: very good Confirmatory Factor Analysis: χ2/df: adequate fit RMSEA: good fit GFI: poor fit AGFI: poor fit Internal consistency: Cronbach's alpha coefficient: good
Ahmadi [39]	Iran	Persian	Female undergraduate students of the Tonekabon branch of Azad Islamic University from six departments, four to five classes were included. Participants who presented losses in three or more items of the questionnaire were excluded	598	21.5 ± 3.4	Female	Eating Attitudes Test-26	Self-reported	26	Diver for thinness Restrained eating Perceived social pressure to eat Food preoccupation and oral Bulimia	Convergent validity: Binge Eating Scale: moderate Beck Depression Inventory-II: null correlation Beck Anxiety Inventory: weak correlation Discriminant validity: Individuals who are currently on a diet Individuals who have never been on a diet statistically significant difference Construct validity: Exploratory factor analysis: Bartlett's test: statistically significant KMO: good Factor Loadings: minimum to high level Internal consistency: Polychoric ordinal alpha: acceptable to excellent Test–retest: Pearson correlation coefficient: null to strong correlation
Pinheiro [48]	Brazil	Portuguese	Preadolescents aged between 8 to 12 years from 4 private schools in São Luis Maranhão were included	347	10.0 ± 1.4	Female/Male	Children's Attitudes Test	Self-reported	26	Dieting Attitudes Oral Control Social pressure to eat	Construct validity: Exploratory factor analysis: Bartlett’s test: statistically significant KMO: average Factorial Loadings: minimum to high level Internal consistency Cronbach's alpha coefficient: moderate

*AN* Anorexia Nervosa, *AUC* Area Under the Curve, *BN* Bulimia Nervosa, *CFI* Comparative Fit Index, *DSM-IV* Diagnostic and Statistical Manual of Mental Disorders-IV, *EDs* Eating Disorders, *HIV* human immunodeficiency virus, *ICC* Intraclass Correlation Coefficient, *SD* Standard deviation, *KMO* Kaiser Meyer Olkin, *TLI* Tucker Lewis Index, *SRMSR* Standardized Root Mean Square Residual, *RMSEA* Root Mean Square Error of Approximation

### Results of individual studies

#### Eating disorder examination (EDE)

The EDE is a semi-structured interview. There are three versions. The first version (12th edition) consisted of 62 items [10], the second version (16th edition) has 41 items [53], and the newest one (17th edition) was created based on the latest Diagnostic and Statistical Manual of Mental Disorders-5 (DSM-5) and has 40 items [54]. All versions contemplate four domains: restraint, eating concern, shape concern, and weight concern. The global EDE score represents the average for the four domains.

The 12th edition of the EDE was translated into Mandarin and the psychometric properties were evaluated by one study in China. The psychometric properties of the Chinese version of the EDE were assessed in a sample of 84 female and male participants, with a mean age of 19 years old. The participant inclusion criteria included having a current diagnosis of AN or bulimia nervosa (BN), according to the Diagnostic and Statistical Manual of Mental Disorders-IV (DSM-IV) criteria, and being admitted to the Wuhan Hospital for psychotherapy or Wuhan Mental Health Center for treatment. The control group consisted of undergraduate and graduate students from the China University of Geosciences in Wuhan who did not have any EDs, according to the author’s interview.

**Fig. 2:**
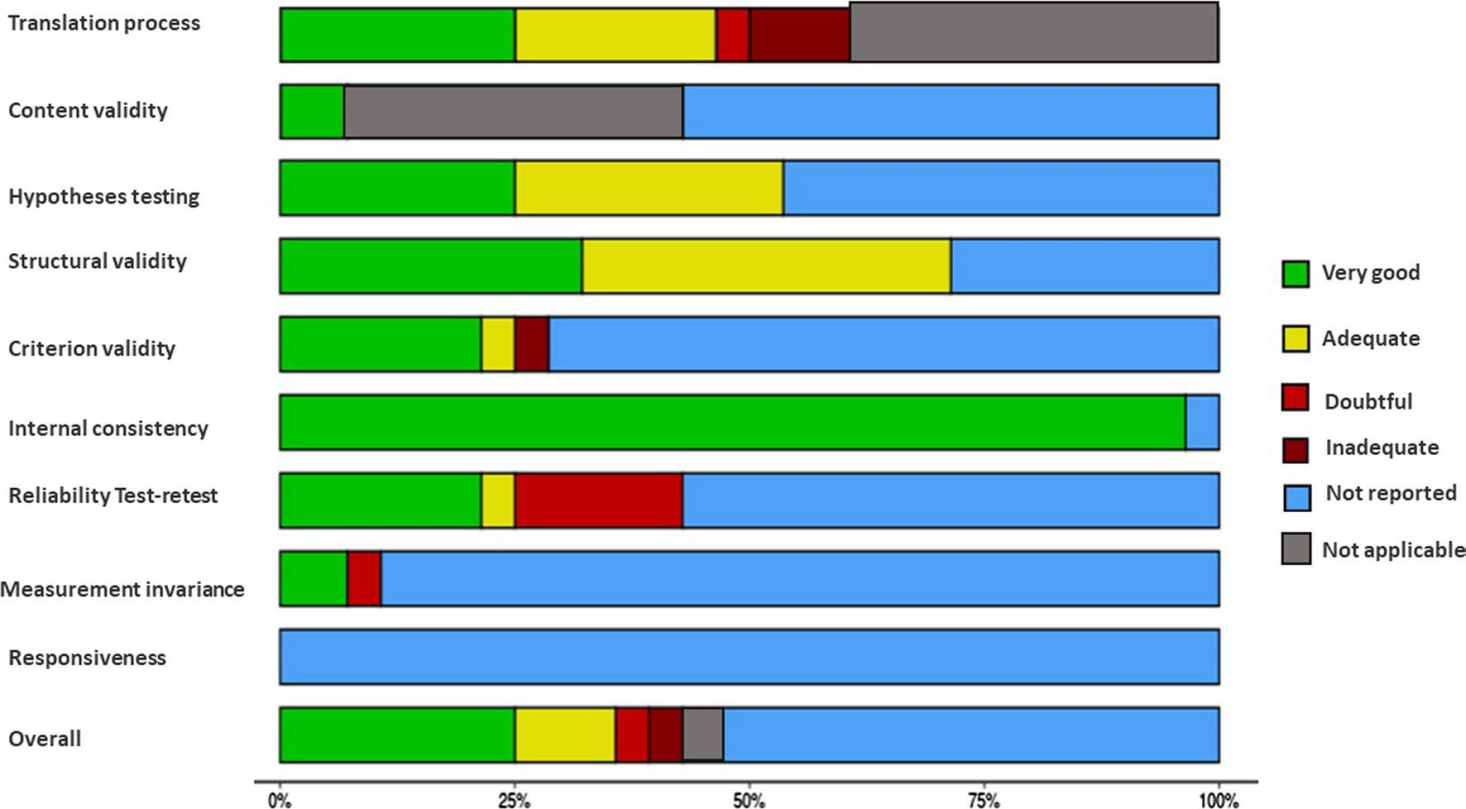
COSMIN methodological quality classification of individual studies. The X-axis represents the percentage of identified studies. *Note* hypotheses testing considers criterion validity; structural validity considers construct validity; and criterion validity considers diagnostic performance

According to the COSMIN methodological quality classification of individual studies the translation process was considered “doubtful”; the hypotheses testing was considered “adequate”; the validity criterion was considered “adequate”; the internal consistency was considered “very good”; and the test–retest assessment was evaluated as “doubtful.” The content validity, structural validity, measurement invariance, and responsiveness were not assessed (see Fig. 2 and Additional file 3) [29]. The EDE instrument met the minimum psychometric criteria considered in this review, through the diagnostic performance evaluation.

The psychometric property results according to the described cut-off points were as follows: discriminant validity: the EDE showed a significant difference between individuals with and without EDs; diagnostic performance: the area under the curve (AUC) was not evaluated; internal consistency: the Cronbach’s alpha coefficient was good; and test–retest: the Spearman correlation coefficient showed a strong correlation [29] (see Table 2).

**Table 2:** Methodology and results of the validation process of the studies

First author (Publication year)	Validity methodology	Reliability	Validation results	Reliability results
Tong [29]	Discriminant validity: Anorexia Nervosa (AN) Bulimia Nervosa (BN) Individuals without Eating Disorders (EDs) Method: Two-tailed independent sample t-test (Mean, Standard Deviation, SD) Diagnostic performance: Anorexia Nervosa (AN) Bulimia Nervosa (BN) Method: Sensitivity Specificity Positive Predictive Values (PPV) Negative Predictive Values (NPV)	Internal consistency: Method: Cronbach's alpha coefficient Test–retest: Method: Spearman coefficient	Discriminant validity: AN: Mean: 3.66; SD: 1.30 BN: Mean: 3.60; SD: 1.30 Individuals without EDs: Mean: 0.43; SD: 0.45 *P* < 0.01 Diagnostic performance: AN Sensitivity: 94% Specificity: 100% PPV: 100% NPV: 96% BN Sensitivity: 96% Specificity 100% PPV: 100% NPV: 94%	Internal consistency: Cronbach's alpha coefficient: 0.89 Test–retest: Spearman coefficient r: 0.88; *p* < 0.01
Penelo [35]	Convergent validity: Presented by group: Women Men Questionnaire on Influences of Aesthetic Body Ideal Eating Disorder Inventory-2 (EDI-2) Children Eating Attitudes Test (ChEAT) Method: Pearson correlation coefficient Construct validity: Confirmatory factor analysis: Method: Maximum likelihood estimation Chi-square statistic/degrees of freedom (χ2/df) Root Mean Square Error of Approximation (RMSEA) Confidence Interval (CI) Comparative Fit Index (CFI) Factor Loading Measurement Invariance Model across Sex Area of residence Method: Equal factor loadings Item intercepts Multiple-Indicator Multiple-Cause (MIMC) Chi-square statistic/degrees of freedom (χ2/df) Root Mean Square Error of Approximation (RMSEA) Confidence Interval (CI) Comparative Fit Index (CFI)	Internal consistency: Method: Omega coefficient Test–retest: Method: Intraclass correlation coefficient Cohen’s Kappa coefficient	Convergent validity: Women: Questionnaire on Influences of Aesthetic Body Ideal: r: 0.75 EDI-2: r: 0.71 ChEAT: r: 0.53 p < 0.01 Men: Questionnaire on Influences of Aesthetic Body Ideal: r: 0.43 EDI-2: r: 0.18 ChEAT: r: 0.28 p < 0.01 Construct validity: Confirmatory factor analysis: χ2/df: 1945.85; 205 RMSEA: 0.05 CI 90%: 0.052; 0.056 CFI: 0.89 Factor loadings: All items with factor loadings > 0.30; p < 0.01 Measurement Invariance: Model equal factor loading: χ2/df: 2887.616; 880 RMSEA: 0.056 CI90%: 0.054; .058 CFI: 0.875 Model equal intercepts χ2/df: 3065.523; 940 RMSEA: 0.056 CI 90%: 0.053; 0.058 CFI: 0.867	Internal consistency: Omega coefficient: 0.94 Test–retest: Intraclass correlation coefficients: 0.84 Cohen’s Kappa: 0.56
Becker [32]	Convergent validity: Fijian Body Shape Concern and Dissatisfaction Questionnaire (FBSQ) Questions on Tradition and Change Global School-Based Student Health Survey (GSHS) Method: Pearson correlation coefficients Construct validity: Exploratory factor analysis: Method: Extraction: Principal axis factoring Rotation: Oblique ProMax Total Variance Factor Loading	Internal consistency: Method: Cronbach's alpha coefficients Test–retest: Method: Intraclass correlation coefficients Cohen’s kappa coefficient	Convergent validity: FBSQ: r: 0.53 Questions on Tradition and Change: r: 0.11–0.23 GSHS: r: 0.27–0.41 *p* < 0.001 Construct validity Exploratory factor analysis: Total Variance: Four factors represent 42% of the total variance Factor loadings: All items with factor loadings > 0.50, range: 0.7–0.8	Internal consistency: Cronbach's alpha coefficient: Range: 0.66 to 0.81 Test–retest: Intraclass correlation coefficients: 0.50–0.70 Cohen’s kappa coefficient: k: 0.13 to 0.81
Mahmoodi [34]	Convergent validity: Clinical Impairment Assessment (CIA) Binge Eating Scale (BES) Method: Pearson correlation coefficient Discriminant validity: Underweight students Overweight student Healthy weight student Method: Univariate Analysis of Variance (Mean, Standard Deviation (SD)	Internal consistency: Method: Cronbach's alpha coefficients	Convergent validity: CIA: r: 0.34 BES: r: 0.60 *p* < 0.01 Discriminant validity: Underweight students: Mean: 0.46; SD: 0.65 Overweight student: Mean: 2.35; SD: 1.27 Healthy weight student: Mean: 1.19; SD: 1.06 *p* < 0.0001	Internal consistency: Cronbach's alpha coefficient: 0.91
Lewis-Smith [28]	Convergent validity: Presented by group: Boys Girls Body Esteem Scale for Adolescents and Adult (BESAA) Method: Spearman's rank correlation coefficient (rho) Construct validity: Exploratory factor analysis: Method: Rotation: Oblique Guttman-Kaiser Criterion Parallel analysis criterion Factor Loadings Relative Chi-Square (Relative χ2) Root Mean Square Error of Approximation (RMSEA) Comparative Fit Index (CFI) Tucker-Lewis Index (TLI) Standardized Root Mean Square Residual (SRMSR) Confirmatory factor analysis: Method: Relative Chi-Square (Relative χ2) Root Mean Square Error of Approximation (RMSEA) Comparative Fit Index (CFI) Tucker-Lewis Index (TLI) Standardized Root Mean Square Residual (SRMSR) Measurement Invariance Boys Girls Method: Multiple Indicators Multiple Causes Models (MIMIC)	Internal consistency: Method: Cronbach's alpha coefficients Test–retest: Method: Boys Girls Cohen’s kappa coefficient	Convergent validity: Boys BESAA: rho: 0.61 *p* < 0.01 Girls BESAA: rho: 0.64 *p* < 0.01 Construct validity: Exploratory factor analysis: Boys: Guttman-Kaiser Criterion: Two factors with eigenvalues > 1; range: 1.92 and 8.98 Parallel Analysis Criterion: Two factors for the solution Factor Loadings: All items with factor loadings > 0.5 Relative χ2: 2.53 RMSEA: 0.06 CFI: 0.95 TLI: 0.93 SRMR: 0.05 Girls: Guttman-Kaiser Criterion: Three factors eigenvalues with > 1; range: 1.4, 2.4 and 9.96 Parallel Analysis Criterion: Two factors for retention Factor Loadings: All items with factor loadings > 0.5 Relative χ2: 2.16 RMSEA: 0.06 CFI: 0.93 TLI: 0.90 SRMR: 0.04 Confirmatory factor analysis: Relative χ2: 5.547^5^ RMSEA: 0.06 CFI: 0.95 TLI: 0.94 SRMR: 0.03 Measurement Invariance Boys and girls MIMIC: 0.244 to 0.317	Internal consistency: Cronbach's alpha coefficient: 0.91 Test–retest: Cohen’s kappa coefficient: Range: 0.25–0.74
Unikel Santoncini [30]	Construct validity: Confirmatory factor analysis: Method: Maximum likelihood Chi-square statistic/degrees of freedom (χ2/df) Root Mean Square Error of Approximation (RMSEA) Comparative Fit Index (CFI) Tucker-Lewis Index (TLI) Standardized Root Mean Residual (SRMR) Factor Loading	Internal consistency: Method: Cronbach's alpha coefficient	Construct validity: Confirmatory factor analysis: χ2/df: 39; 11; p < 0.001 RMSEA: 0.07; 90% CI: 0.05, 0.10 CFI: 0.99 TLI: 0.98 SRMR: 0.02 Factor Loadings: All items with factor loadings > 0.60; range: 0.66–0.91	Internal consistency: Cronbach's alpha coefficient: 0.9
He [26]	Convergent validity: Eating Attitude Test‑26 (EAT-26) Kessler Psychological Distress Scale (K10) Method: Pearson correlation coefficients Construct validity: Confirmatory factor analysis: Method: Chi-square statistic/degrees of freedom (χ2/ df) Comparative Fit Index (CFI) Tucker-Lewis Index (TLI) Root Mean-Square Error of Approximation (RMSEA) with (Confidence Interval 90% CI) Factor Loadings Item response theory: Rasch modeling: Method: Unidimensionality: Principal components analysis: Variance Explained Unexplained Variance Eigenvalue Item calibration: Information weighted ft statistic (inft) Outlier-sensitive ft statistic (outfit) Person separation index Person separation reliability Differential Item Functioning (DIF)	Internal consistency: Method: Cronbach's alpha coefficient Test–retest: Method: Intraclass correlation coefficient	Convergent validity: EAT-26: r: 0.56 K10: r: 0.44 *p* < 0.01 Construct validity: Confirmatory factor analysis: χ2/df: 1060.34; 54, *p* < 0.01 CFI: 0.93 TLI: 0.91 RMSEA: 0.14; 90% CI: 0.13–0.14 Factor Loadings: All items with factor loadings > 0.6 Item response theory: Rasch modeling: Unidimensionality: Principal components analysis Variance Explained: 48.9% Unexplained Variance: 9.6% Eigenvalue: 2.25 Item calibration Inft: Range: 0.89 to 1.28 Outfit: Range: 0.84 to 1.31 Person separation index: 2.17 Person separation reliability: 8.13 DIF: Contrast value for item 6 0.67, the rest of the items < 0.55 logits	Internal consistency: Cronbach's alpha coefficient: 0.89 Test–retest: Intraclass correlation coefficient: 0.82
Yucel [31]	Convergent validity: Eating Attitudes Test (EAT) Turkish version of the General Health Questionnaire (GHQ) Body Image Satisfaction Method: Pearson correlation coefficient	Internal consistency: Method: Cronbach's alpha coefficients Test-rest: Method: Pearson correlation coefficient	Convergent validity: EAT: r: 0.49 Turkish version of the GHQ: r: 0.41 BIS: r: −0.25 *p* < 0.001	Internal consistency: Cronbach's alpha coefficient: 0.93 Test–retest: Pearson correlation coefficient: r: 0.91; *p* < 0.001
Ramli [36]	Construct validity: Confirmatory factor analysis: Method: Rotation: Varimax Keiser value Eigenvalues Total Variance Factor loading	Internal consistency: Method: Cronbach's alpha coefficients	Construct validity: Confirmatory factor analysis: Keiser value: 0.89 Eigenvalues: Four factors with eigenvalues > 1 Total Variance: Four factors explained 59% of the total variance Factor loading: 21 of the 26 items with factor loadings < 0.3; range: 0.3–0.8	Internal consistency: Cronbach's alpha coefficient: 0.87
Mohd Taib [27]	Convergent validity: Eating Attitudes Test (EAT) World Health Organization Quality of Life Brief (WHOQL) Method: Pearson correlation coefficient Construct validity: Exploratory factor analysis: Method: Extraction: principal axis factoring Rotation: Oblique Eigenvalue Total variance Factor loadings	Internal consistency: Method: Cronbach's alpha coefficient Test–retest: Method: Pearson correlations coefficient	Convergent validity: EAT: r: 0.53 WHOQL: r: 0.27 *p* < 0.01 Construct validity: Exploratory factor analysis: Eigenvalue: Four factors with eigenvalue > 1; range 1.1–9.4 Total variance: Four factors explained 63% of the total variance Factor loadings: 27 of the 28 items with factor loadings in all factors < 0.3; range: 0.3–0.9	Internal consistency: Cronbach's alpha coefficient: 0.93 Test–retest: Pearson correlation: coefficient: r: 0.83 *p* < 0.01
Compte [33]	Construct validity: Confirmatory factor analysis: Presented by group: College students Weightlifter Cross-fit Rugby players Method: Robust maximum likelihood estimation Chi-square statistic/degrees of freedom (χ2/df) Comparative Fit Index (CFI) Tucker–Lewis Index (TLI) Standardized Root Mean Square Residual (SRMSR) Root Mean Square Error of Approximation (RMSEA); (Confidence Interval 90% (CI) Measurement Invariance Configural invariance Metric invariance Scalar invariance Method: Δ Comparative Fit Index (CFI) Δ Root Mean Square Error of Approximation (RMSEA) Δ Standardized Root Mean Square Residual (SRMR)	Internal consistency: Presented by group: College students Weightlifter Cross-fit Rugby players Method: Omega coefficients (Confidence Interval 95% (CI)	Construct validity: Confirmatory factor analysis: College students: χ2/df: 2.17 CFI: 0.96 TLI: 0.94 SRMSR: 0.04 RMSEA: 0.07; CI 90%: 0.05, 0.09 Weightlifters: χ2/df: 2.55 CFI: 0.94 TLI: 0.92 SRMSR: 0.04 RMSEA: 0.08; CI 90%: 0.05, 0.10 Cross fit: χ2/df: 1.82 CFI: 0.95 TLI: 0.93 SRMSR: 0.05 RMSEA: 0.06; CI 90%: 0.04, 0.10 Rugby: χ2/df: 2.57 CFI: 0.91 TLI: 0.87 SRMSR: 0.05 RMSEA: 0.09; CI 90%: 0.06, 0.11 Measurement Invariance Configural vs Metric: ΔCFI: 0.003 ΔRMSEA: 0.008 ΔSRMR: 0.024 Metric vs Scalar: ΔCFI: 0.003 ΔRMSEA: 0.005 ΔSRMR: 0.002	Internal consistency: Omega coefficients: College students: 0.91; CI 95%: 0.88, 0.93 Weightlifter: 0.86; CI 95%: 0.82, 0.89 Cross-fit: 0.86; CI 95%: 0.82, 0.91 Rugby players: 0.86; CI 95%: 0.82, 0.90
Unikel Santoncini [37]	Convergent validity: Symptom checklist Coopersmith Self Esteem Inventory (CSEI) Method: Pearson correlation coefficient Construct validity: Exploratory factor analysis: Method: Extraction: Principal components analysis Rotation: Varimax Eigenvalues Total Variance Factor Loadings	Internal consistency: Method: Cronbach's alpha coefficients	Convergent validity: Symptom check list: r: 0.45–0.71 CSEI: r: −0.63 *p* < 0.01 Construct validity: Exploratory factor analysis: Eigenvalue: Six factors with eigenvalues > 1; range: 1.2–9.9 Total Variance: Six factor explained 56% of the total variance Factor Loadings: All items with factor loadings > 0.50; range: 0.51–0.80	Internal consistency: Cronbach's alpha coefficient: 0.93
García-García [38]	Diagnostic performance: Method: Sensitive cut-off point Sensitivity (Confidence Interval 95% CI) Specificity (Confidence Interval 95% CI) Positive Predictive Value (PPV) Negative Predictive Value (NPV) Specific cut-off point	Internal consistency: Method: Cronbach's alpha coefficient	Diagnostic performance: Sensitive cutoff point: 80% Sensitivity: 91%; CI 95%: 69 a 98 Specificity: 80%; CI 95%: 58 to 92 PPV: 82% NPV: 87% Specific cutoff point: 105% Sensitivity: 81%; CI 95%: 59 to 94 Specificity: 89%; CI 95%: 70 to 97 PPV: 85% NPV: 84%	Internal consistency: Cronbach's alpha coefficient: 0.85
Dadgostar [40]	Content validity: Method: Content Validity Index Validity index for clarity Validity index for relevancy Comprehensiveness of the survey	Internal consistency: Method: Cronbach's alpha coefficient Test–Retest: Method: Intraclass correlation coefficient	Content validity: Validity index for clarity: 0.91; 89 items out of 91 Validity index for relevancy: 0.89; 87 items out of 91 Comprehensiveness of the survey: 100%	Internal consistency: Cronbach's alpha coefficients: Range: 0. 6–0.8 Test–retest: Intraclass correlation coefficient: Range: 0.69–0.71
Rutsztein [39]	Construct validity: Exploratory factor analysis: Eating Disorder Risk Scale Scales of psychological characteristics Method: Extraction: Maximum likelihood Rotation: Promax Bartlett's test Kaiser–Meyer–Olkin coefficient (KMO) Eigenvalues Total Variance	Internal consistency: Method: Cronbach's alpha coefficient	Construct validity: Exploratory factor analysis: Eating Disorder Risk Scale: Bartlett's test: χ^2^: 902.567 g.l.: 300; *p* < 0.01 KMO: 0.93 Eigenvalue: Three factors with eigenvalue > 1; range: 1.6–9.047 Total Variance: Three factor explained 53% of the total variance Scales of psychological characteristics: Bartlett's test: χ^2^: 16,851.928; g.l.: 208; *p* < 0.01 KMO: 0.91. Eigenvalues: Eight factors with eigenvalue > 1; range: 1.5–12.39 Total Variance: Eight factor explained 44% of the total variance	Internal consistency: Cronbach's alpha coefficient: Range: 0.63–0.97
Savaşır [25]	Construct validity: Exploratory factor analysis: Method: Extraction: principal component analysis Total variance Factor loadings	Internal Consistency: Method: Cronbach's alpha coefficient	Construct validity: Exploratory factor analysis: Total variance: Four factors explained 59% of the total variance Factor loadings: 13 of the 40 items with factor loadings > 0.30; range: 0.35–0.73	Internal consistency: Cronbach's alpha coefficient: 0.70
Nasser [41]	Construct validity: Confirmatory Factor Analysis: Method: Orthogonal structure Rotation: Varimax Screen Test with eigenvalues Total Variance Factor Loadings	Internal consistency: Internal validity of each factor: Factor dieting Factor bulimia and food preoccupation Factor oral control Method: Cronbach's alpha coefficient	Construct Validity: Confirmatory Factor Analysis: Scree plot with eigenvalues: Three factors with eigenvalues > 1; range:1.01–3.54 Total variance: Three factors explained 54,2% of the total variance Factor loadings: 15 of the 26 items presented factor loadings > 0.40; range: 0.40–0.99	Internal consistency: Cronbach's alpha coefficient: Range: 0.2–0.8
Alvarez-Rayón [44]	Discriminant validity: Individuals with Eating Disorders (EDs) Control Group Method: Mean, Standard Deviation SD Construct validity: Exploratory factor analysis: Method: Rotation: Varimax Criterion of Eigenvalue Total Variance Factor Loadings Diagnostic performance: Method: Cut-off Sensitivity Specificity Positive Predictive Value (PPV) Negative Predictive Value (NPV)	Internal consistency: Method: Cronbach's alpha coefficient	Discriminant validity: Individuals with EDs: Mean: 49.1; SD: 11.3 Control Group: Mean: 15.5; SD: 5.0 *p* ≤ 0.0001 Construct validity: Exploratory factor analysis: Criterion of Eigenvalue: Five factors with eigenvalue > 1; range: 1.5–9.4 Total Variance: Five factors explained 46% of the total variance Factor Loadings: 25 of the 40 items with factor loadings > 0.30; range: 0.41–0.84 Diagnostic performance: Cut-off: 26 Sensitivity: 83% Specificity: 91% PPV: 16.3% NPV: 9.3%	Internal consistency: Cronbach's alpha coefficient: 0.93
Nunes [45]	Diagnostic performance: Method: Cut-off point Sensitivity Specificity Positive Predictive Value (PPV) Negative Predictive Value (NPV)	Internal consistency: Method: Cronbach's alpha coefficient	Diagnostic performance: Cut-off point ≥ 21 Sensitivity: 40% Specificity: 84% PPV: 14% NPV: 95%	Internal consistency: Cronbach's alpha coefficient: 0.75
Fortes [46]	Convergent validity: Body Shape Questionnaire (BSQ) Method: Spearman Rank correlation Discriminant validity: Anthropometric data: Low weight Normal weight Overweight Obese Method: ANOVA one-way (Mean; Standard Deviation, (SD) Construct validity: Exploratory factor analysis Method: Extraction: Principal components analysis Rotation: Oblimin Bartlett's Test Kaiser–Meyer–Olkin coefficient (KMO) Total Variance Factor Loadings	Internal consistency: Method: Cronbach's alpha coefficient Test–retest: Method: Intraclass correlation coefficient	Convergent Validation: BSQ: r-spearman: 0.50 *p* < 0.01 Discriminant validaty: Low weight: Mean: 10.5; SD: 3.9 Normal weight: Mean: 13.4; SD: 1.2 Overweight: Mean: 14.8; SD: 2.6 Obese: Mean: 21.1; SD: 4.2 *p* < 0.05 Construct validity: Exploratory factor analysis: Bartlett's Test: 3567; *p* < 0.01 KMO: 0.92 Total Variance: A single factor was responsible for explaining 32.8% of the total variance. Factor Loadings: 24 of the 26 items with factor loadings > 0.30-range: 0.34–0.73	Internal consistency: Cronbach's alpha coefficient: 0.88 Test–retest: Intraclass correlation coefficient: 0.93; *p* < 0.01
Kang [48]	Convergent validity Eating Disorder Inventory (EDI) Method: Pearson correlation coefficient Diagnostic performance: Method: A receiver operating characteristic Area under the curve (AUC) Sensitivity Specificity Youden Index (YI)	Internal consistency: Method: Cronbach's alpha coefficient Test–retest: Method: Interclass correlation coefficient	Convergent validity: EDI: r: 0.45–0.75 *p* < 0.001 Diagnostic performance: Cut-off: 15 AUC: 0.83; 95% CI: 0.80–0.81 Sensitivity: Range: 0.66 to 0.68 Specificity: Range: 0.85–0.86 YI: 0.52	Internal consistency: Cronbach's alpha coefficient: Range: 0.82–0.92 Test–retest: Interclass correlation coefficient: 0.81
Constaín [49]	Construct validity: Exploratory factor analysis: Method: Extraction: Principal components analysis Rotation: Varimax Correlation matrix Bartlett's test Kaiser–Meyer–Olkin coefficient (KMO) Eigenvalue Total variance Factorial loadings Diagnostic performance: Method: ROC Curves (receiver operating characteristic) Area under the curve (AUC) Sensitivity Specificity Positive Predictive Value (PPV) Negative Predictive Value (NPV) + Likelihood Ratio (+ LR)—Likelihood Ration (-LR) (Confidence Interval 95% CI)	Internal consistency: Method: Cronbach's alpha coefficient	Construct validity: Exploratory factor analysis: Correlation matrix: 0.000000291 Bartlett's test: 2.46.48; *p* < 0.0001 KMO: 0.90 Eigenvalue: Four factors with > 1 Total Variance: Four factors represent 66% of the total variance. Factorial loadings: All items with factorial loadings > 0.50; range: 0.55 to 0.85 Diagnostic performance: Cut-off value ≥ 11 AUC: 97.3% (z = 20.7, p < 0,0001) Sensitivity: 100%; CI 95%: 86.3–100% Specificity: 85.6%; CI 95%: 77.6–91.5% PPV: 61%; CI 95%: 44.5–75.8 NPV: 100%; CI 95%: 96.2–100.0% + LR: 6.9%; CI 95%: 4.4–10.9%-LR: 0.0%	Internal consistency: Cronbach's alpha coefficient: 0.92
Constaín [50]	Construct validity: Exploratory factor analysis: Method: Extraction: Principal components analysis Rotation: Varimax Bartlett's test Kaiser–Meyer–Olkin coefficient (KMO) Eigenvalue Total variance Factor loadings Diagnostic performance: Method: ROC Curves (receiver operating characteristic) Area under the curve (AUC Sensitivity Specificity Positive Predictive Value (PPV) Negative Predictive Value (NPV) + Likelihood Ratio (+ LR)—Likelihood Ration (-LR) (Confidence Interval 95% CI)	Internal consistency: Method: Cronbach's alpha coefficient	Construct validity: Exploratory factor analysis: Bartlett's test: 325; *p* < 0.0001 KMO: 0.78 Eigenvalue: Four factors with eigenvalue > 1 Total variance: Four factors represent 56.4% of the total variance. Factor loadings: All items with factor loadings > 0.50; range: 0.44–0.79 Diagnostic performance: Cut-off value ≥ 21 AUC: 99.9% (z = 142.3; p < 0,0001) Sensitivity: 100%; CI 95%: 86.3–100% Specificity: 85.6%; CI 95%: 79.3–100% PPV: 93%; CI 95%: 72.0 -98.9% NPV: 100%; CI 95%: 96.0–100.0% + LR: 46.5%; CI 95%: 45.1–47.9% -LR: 0.0%	Internal consistency: Cronbach's alpha coefficient: 0.89
Erguney-Okumus [51]	Convergent validity: Eating Attitude Test-40 (EAT-40) Brief Symptom Inventory (BSI) Eating Disorder Rating Scale Method: Pearson correlation coefficient Construct validity: Exploratory Factor Analysis: Method: Rotation: Oblique Bartlett's test Kaiser–Meyer–Olkin coefficient (KMO) Eigenvalue Total variance Factor loadings Confirmatory factor analysis: Method: Robust maximum likelihood estimation Chi-square statistic/degrees of freedom (χ2/df) Comparative Fit Index (CFI) Goodness-of-Fit Index (GFI) Standardized Root Mean Square Residual (SRMSR) Root Mean Square Error of Approximation (RMSEA)	Internal consistency: Method: Cronbach's alpha coefficient Test–retest: Method: Pearson correlation coefficient	Convergent validity: EAT-40: r: 0.48 BSI: r: 0.22 Eating Disorder Rating Scale: r: 0.65 *p* < 0.001 Construct validity: Exploratory factor analysis: Bartlett's test: 325; *p* < 0.001 KMO: 0.88 Eigenvalue: Three factors with eigenvalues > 1 Total variance: Three factors explained 38.5% of the total variance Factor loadings: All items with factor loadings > 0.30; range: 0.31 to 0.80 Confirmatory factor analysis: χ2/df: 2.92 CFI: 0.84 GFI: 0.89 SRMR: 0.08 RMSEA: 0.07	Internal consistency: Cronbach's alpha coefficient: Range: 0.62–0.85 Test–retest: Pearson correlation coefficient: r: 0.78; *p* < 0.001
Kaewporndawan [47]	Content validity: Method: Content validity index Item total correlation coefficient Discriminant validity: Individuals with Eating Disorders (EDs) Control group Method: (Independent sample test-t); Mean, Standard Deviation, SD Diagnostic performance: Method: Area Under the Curve (AUC) Cut-off Sensitivity Specificity Positive Predictive Value (PPV) Negative Predictive Value (NPV) Positive Likelihood Ratio (+ LR) Negative Likelihood Ratio (-LR)	Not Applicable	Content validity: Item total correlation coefficient: 25 of the 26 items were > 0.5, except for question 26 of 0.82 Discriminant validity: Individuals with EDs: Mean: 30.4; SD: 15.7 Control group: Mean: 6.5; SD: 5.9 *p* < 0.001 Diagnostic performance: AUC: 0.93 Cut-off point: 12 Sensitivity: 71% Specificity: 94% PPV: 92% NPV: 76% + LR: 11.8 -LR: 0.31	Not Applicable
Haddad [42]	Construct validity: Exploratory factor analysis: Method: Extraction: principal component analysis Rotation: Promax Bartlett's test of sphericity Kaiser–Meyer–Olkin coefficient (KMO) Eigenvalue Total Variance Confirmatory factor analysis: Method: Maximum likelihood Relative chi-square (χ2/df) Root Mean Square Error of Approximation (RMSEA) Goodness of Fit Index (GFI) Adjusted Goodness of Fit Index (AGFI)	Internal consistency: Method: Cronbach's alpha coefficient	Construct validity: Exploratory factor analysis: Bartlett's test of sphericity: *p* < 0·001 KMO: 0·91 Eigenvalue: Six factors with eigenvalues > 1 Total variance: Six factors explained 60% of the total variance Confirmatory Factor Analysis: Method: Maximum likelihood χ2/df: 2.4 RMSEA: 0.13 GFI: 0.76 AGFI: 0.71	Internal consistency: Cronbach's alpha coefficient: 0.89
Ahmadi [43]	Convergent validity: Binge Eating Scale (BES) Beck Depression Inventory-II (BDI-II) Beck Anxiety Inventory (BAI) Method: Bonferroni correction Discriminant validity: Method: Individuals who are currently on a diet Individuals who have never been on a diet Method: Multivariate analysis of variance (Mean; Standard Deviation; SD) Construct validity: Exploratory factor analysis: Method: Extraction: Principal component analysis Rotation: Varimax Bartlett's test Kaiser–Meyer–Olkin coefficient (KMO) Eigenvalue Total Variance Factor Loadings	Internal consistency: Method: Polychoric ordinal alpha Test–retest: Method: Pearson correlation coefficient	Convergent validity: BES: r: 0.46 BDI-II: r: 0.19 BAI: r: 0.26 *p* < 0.001 Discriminant validity: Individuals who are currently on a diet: Mean: 20.4; SD: 1.1 Individuals who have never been on a diet: Mean: 8.8; SD: 0.5 *p* < 0.001 Construct validity: Exploratory factor analysis: Bartlett's test: 3.977.12; *p* < 0.0001 KMO: 0.82 Eigenvalue: Five factors with eigenvalue > 1; range 1.26–5.7 Total Variance: Five factors represent 50% of the total variance. Factor Loadings: All items with factor loadings > 0.30; range: 0.33–0.87	Internal consistency: Polychoric ordinal alpha: Range: 0.76–0.92 Test–retest: Pearson correlation coefficient: Range: 0.26–0.64
Pinhero [52]	Construct validity: Exploratory factor analysis: Method: Extraction: Principal components analysis Rotation: Varimax Bartlett’s test Kaiser–Meyer–Olkin coefficient (KMO) Scree plot with eigenvalues Total Variance Factor Loadings	Internal consistency: Method: Cronbach's alpha coefficient	Construct validity: Exploratory factor analysis: Bartlett’s test: 0.003 KMO: 0.70 Scree plot with eigenvalues: Three factors with eigenvalues > 1 Total Variance: Three factors explained 33% of the total variance Factorial Loadings: All items with factor loadings > 0.30; range: 0.35–0.81	Internal consistency: Cronbach's alpha coefficient: 0.69

*AGFI* Adjusted Goodness of Fit Index, *AN* Anorexia Nervosa, *AUC* Area Under the Curve, *BAI* Beck Anxiety Inventory, *BDI-II* Beck Depression Inventory-II, *BES* Binge Eating Scale, *BSI* Brief Symptom Inventory, *BESAA* Body Esteem Scale for Adolescents and Adult, *BN* Bulimia Nervosa, *ChEAT* Children Eating Attitudes Test, *CIA* Clinical Impairment Assessment, *CI* Confidence Interval, *CFI* Comparative Fit Index, *DIF* Differential Item Functioning, *EAT-40* Eating Attitude Test-40, *EDs* Eating disorders, *EDI-2* Eating Disorder Inventory-2, *FBSQ* Fijian Body Shape Concern and Dissatisfaction Questionnaire, *GFI* Goodness of Fit Index, *GSHS* Global School-Based Student Health Survey, *KMO* Kaiser–Meyer–Olkin coefficient, *K10* Kessler Psychological Distress Scale*, + LR* Positive Likelihood Ratio, *−LR* Negative Likelihood Ratio, *MIMC* Multiple Indicators Multiple Causes Models, *NPV* Negative Predictive Value, *PPV* Positive Predictive Value, *RMSEA* Root Mean Square Error of Approximation, *ROC* receiver operating characteristic, *SD* Standard Deviation, SRMSR Standardized Root Mean Square Residual, *TLI* Tucker–Lewis Index, outfit Outlier-sensitive ft statistic, χ2/df Relative chi-square χ2/df Chi-square statistic/degrees of freedom; inft Information weighted ft statistic, *WHOQL* World Health Organization Quality of Life Brief, *YI* Youden Index

#### Eating disorder examination-questionnaire (EDE-Q)

The EDE-Q is a self-report version of the EDE. There are three versions of it: the EDE-Q 4.0 with 38 items [11], the EDE-Q 6.0 with 28 items [55], and the short version EDE-QS with 12 items [56]. The EDE-Q 4.0 and EDE-Q 6.0 have four domains: restraint, eating concern, shape concern, and weight concern. The global EDE-Q score represents the average of the four domains. Finally, the EDE-QS has 12 items and is the only version that is one-dimensional.

The included studies used the three versions of the EDE-Q: the EDE-Q 4.0 [30, 31], the EDE-Q 6.0 [22, 23, 25–29], and the EDE-QS [21]. The EDE-Q was translated by eight studies [26–28, 30–32, 34, 36]. Two did not carry out the translation process and used a version of the questionnaire that was already translated into Spanish [33, 35, 57]. Ten studies evaluated the psychometric properties [26–28, 30–36]. The studies were conducted in the following countries and languages: Mexico (in Spanish) [30, 35], Malaysia (in Malay) [27, 36], Fiji (in Fijian) [32], Argentina (in Spanish) [33], China (in Mandarin) [26], India (in English) [28], Iran (in Persian) [34], and Turkey (in Turkish) [31]. The number of participants in the studies varied from 298 [36] to 2,928 [35]. Six studies were conducted in female and male participants [26–28, 31, 35, 36], three studies in female participants [30, 32, 34], and only one study in male participants [33]. The mean age of the participants varied from 10 [36] to 26 [33] years old. The participant inclusion criteria varied according to the target population chosen in the studies. In one study, all participants were recruited through convenience sampling at a hospital among patients with an eating disorder diagnosis [30], in six studies the participants were chosen through convenience sampling at secondary schools [26, 28, 30–32, 35], in three studies they were recruited at universities [27, 33, 34], and in one study they were recruited at sports centers [33]. In three studies, the exclusion criterion was participants who did not answer three or more questionnaires [24, 30, 31]. One study excluded participants from vernacular schools or schools with a predominant ethnic group [36].

According to the COSMIN methodological quality assessment, the translation process of the EDE-Q was considered “very good or adequate” in 80% of the studies [26–28, 30–32, 34, 36]; the hypotheses testing process was classified as “very good or adequate” in 70% [26–28, 31, 32, 34, 35]; the structural validity was classified as “very good” in 60% [26, 28, 30, 33, 35, 36]; the internal consistency assessment was evaluated as “very good” in 100% [26–28, 30–36]; the test–retest assessment was classified as “very good” in 40% [26, 28, 32, 35] and “doubtful” in 20% [27, 31]; and the measurement invariance was assessed as “very good” in 20% [33, 35] and “inadequate” in 10% of the studies [28]. Content validity, criterion validity, and responsiveness were not described in any of the studies, (see Fig. 2 and Additional file 3). No study contemplated the minimum psychometric assessment (content, criterion, and construct validity) or diagnostic performance.

The psychometric property results according to the described cut-off points were as follows: discriminant validity: the EDE-Q showed a significant difference between individuals who are overweight, underweight, and have a normal weight [34]. Convergent validity showed a null [31] to strong correlation [35]. The construct validity results assessed through CFA were as follows: relative χ2: inadequate [28] to adequate fit [26, 28, 30, 33, 35]; comparative fit index (CFI): unacceptable fit [26, 35] to good fit [28, 30]; Tucker-Lewis index (TLI): unacceptable fit [26, 28, 33] to good fit [30]; root mean square error of approximation (RMSEA): marginal [33] to good fit; standardized root mean residual (SRMR): good fit [28, 30, 33]; factor loadings: minimum [35, 36] to high level [26, 30, 36]; Kaiser–Meyer–Olkin coefficient (KMO): good [36]. The exploratory factor analysis (EFA) results were: KMO and Bartlett’s test not evaluated [27, 32]; factor loadings: minimum [27] to high level [27, 28, 32]; relative χ2: adequate fit [28]; CFI: unacceptable fit [28] to good fit [28]; TLI: unacceptable fit [28]; RMSEA: acceptable [28] to good fit [28]; and SRMR: good fit [28]. The item response theory results were: explained variance: strong [26]; unexplained variance: good [26]; eigenvalue: acceptable [26]; infit: productive [26]; outfit: productive [26]; Person separation index: good [26]; Person separation reliability: good [26]; differential item functioning: intermediate to large [26]. The measurement invariance results were: equal factor loading χ2/df: adequate fit [35]; RMSEA: acceptable fit [35]; CFI: unacceptable adjustment [35]. The model equal intercepts were: χ2/df: inadequate fit [35]; RMSEA: acceptable fit [35]; CFI: unacceptable fit [35]; configural vs metric: ΔCFI: non invariance [33]; ΔRMSEA: non invariance [33]; ΔSRMR: strong invariance [33]; metric vs scalar: ΔCFI: non invariance [33]; ΔRMSEA: non invariance [33]; ΔSRMR: strong invariance [33]. In one study CFI RMSEA, and SRMR were not assessed [28]. The internal consistency results were: omega coefficient: good [33, 35] to excellent [33]; Cronbach’s alpha coefficient: moderate [32] to excellent [28, 30, 31, 34]. The test–retest results were: intraclass correlation coefficients (ICC): good [26, 35]; Cohen’s kappa coefficient: poor [32] to substantial [32]; and Pearson correlation coefficient: strong [27] to very strong [31] (see Table 2).

### Eating disorder inventory (EDI)

The EDI is a self-report questionnaire and has three versions. The first version has 64 items grouped into eight domains: drive for thinness, bulimia, body dissatisfaction, perfectionism, ineffectiveness, interpersonal distrust, interoceptive awareness, and maturity fears [12]; the second version (EDI-2) has 91 items grouped into 11 domains: drive for thinness, bulimia, body dissatisfaction, perfectionism, ineffectiveness, interpersonal distrust, interoceptive awareness, maturity fears, asceticism, impulse regulation, and social insecurity [58]; and the third and most recent version (EDI-3) consists of 91 items distributed in 12 domains: drive for thinness, bulimia, body dissatisfaction, low self-esteem, emotional dysregulation, perfectionism, asceticism, interoceptive deficit, maturity fear, interpersonal insecurity, personal alienation, and interpersonal alienation [59].

The included studies used the three versions of the EDI: the EDI [37], the EDI-2 [38], and the EDI-3 [39, 40]. A translated version of the EDI was used by two studies [38, 40]. These two studies did not carry out the translation process but instead used the version of the questionnaire already available in Spanish [37, 39, 60, 61]. The studies were conducted in Mexico (in Spanish) [37, 38], Iran (in Persian) [40], and Argentina (in Spanish) [39]. The number of participants in the studies varied considerably, from 47 [38] to 725 [39]. Three studies included only female participants [37–39] and one study included female and male participants [40]. The mean age of the participants varied from 15 [39] to 22 [40] years old. The participant inclusion criteria varied according to the target population chosen in the studies. All participants were recruited through convenience sampling. Two studies included patients diagnosed with an eating disorder according to the DSM-IV criteria, who were recruited in hospitals [37, 38]; one study recruited participants in a secondary school setting [39]; and one study recruited participants at a university [40]. The latter study excluded participants who did not answer three or more items in the questionnaires [40], and another study excluded students who presented difficulties with communication and in understanding the questionnaire [39].

According to the COSMIN methodological quality assessment of the four studies, the translation process was considered “adequate” in 25% of the studies [40] and “inadequate” in 25% [38]; the content validation process was considered “very good” in 50% of the studies [35]; the structural validity assessment was “adequate” in 50% of the studies [37, 39]; and the internal consistency examination was considered “very good” in 100% of the studies [37–40]. Hypotheses testing, criterion validity, test–retest, measurement invariance, and responsiveness were not described in any of the studies [38–40] (see Fig. 2 and Additional file 3). Two studies contemplated the minimum psychometric assessment [37, 38], one by examining content, criterion, and construct validity (25%) [37], and the other by evaluating diagnostic performance (25%) [38].

The psychometric property results according to the described cut-off points were as follows: content validity: the validity index for the clarity, relevance, and comprehensiveness of the survey was acceptable [40]; convergent validity: null [37] to strong correlation [37]; construct validity through EFA: KMO: good [39] and Bartlett’s test not evaluated [37, 39]; factor loadings: high level [37]; diagnostic performance: AUC not evaluated [38]; internal consistency: Cronbach’s alpha coefficient: questionable [39, 40] to excellent [37, 39]; and test–retest: moderate ICC [40] (see Table 2).

### Eating attitudes test (EAT)

The EAT is a self-report questionnaire and has two versions. The first version has 40 items [13] and the second and most recent version (EAT-26) has 26 items [62]. Both versions contemplate three domains: dieting, bulimia and food preoccupation, and oral control [13, 62].

The included studies used the two versions of the EAT: the EAT-40 [25, 41, 44] and the EAT-26 [42, 43, 45–51]. The EAT was translated by five studies [25, 42, 43, 47, 51] and seven studies did not carry out the translation process because they used the versions of the questionnaire already translated into Arabic, Spanish, Portuguese, and Mandarin, respectively [41, 44–46, 48–50, 63–66]. All studies evaluated the psychometric properties [25, 41–51]. The studies were conducted in Colombia (in Spanish) [49, 50], Brazil (in Portuguese) [45, 46], Turkey (in Turkish) [25, 51], Egypt (in Arabic) [41], Mexico (in Spanish) [44], Lebanon (in Arabic) [42], China (in Mandarin) [48], Thailand (in Thai) [47], and Iran (in Persian) [43]. The number of participants in the studies varied from 70 [47] to 1500 [51]. In eight studies, the questionnaire was administered to female participants [25, 41, 43–45, 47–49], two studies included only male participants [46, 50], and two studies were conducted in mixed samples of males and females [42, 51]. The mean age of the participants varied from 14 [46] to 27 [42] years old. All participants were recruited through convenience sampling. In five studies, a clinical group was recruited at a hospital for patients with an eating disorder diagnosis [44, 47–50]; in five studies, community participants were recruited from secondary schools [25, 41, 44, 46, 48]; and seven studies were conducted at universities [25, 43, 44, 48–51]. In two studies, the exclusion criterion was participants who did not answer three or more items of the questionnaires [43, 46]. Two studies excluded participants with stupors, depression, catatonia, schizophrenia, neoplasms, any severe medical illness related to malnutrition, and hypometabolism [49, 50]. One study excluded elementary school students [25] and another excluded participants who did not perform anthropometric measurements [46]. One study excluded participants who refused to complete the questionnaire and participants who had a cognitive impairment, as reported by a family member [51].

According to the COSMIN methodological quality assessment, the hypotheses testing was considered “very good or adequate” in 50%of the studies [43, 44, 46–48, 51]; the structural validity examination was classified as “very good or adequate” in 75% of the studies [25, 41–44, 46, 49–51]; the criterion validity assessment was classified as “very good” in 42% of the studies [45, 47–50] and “inadequate” in 8% [44]; the translation process was considered “not applicable” in58% [41, 44–46, 48–50] and of the ones that carried out the translation process, the content validation was “not reported” in 80% [25, 42, 43, 51]; the internal consistency assessment was classified as “very good” in96% [25, 41–46, 48–51]; and the test–retest was “not reported” in67% of the studies [25, 41, 42, 44, 45, 47, 49, 50]. Measurement invariance and responsiveness were not assessed in these studies (see Fig. 2 and Additional file 3). Seven studies contemplated the minimum psychometric assessment [44–50], six by testing diagnostic performance (50%) [44, 45, 47–50], and one study by examining content, criterion, and construct validity (8%) [46].

The psychometric property results according the described cut-off points were: content validity: content validity index: the total item coefficient was excellent [47]; discriminant validity: the EAT showed a significant difference between individuals with and without EDs [44, 47], between individuals who are low weight, normal weight, overweight, and obese [46], and between dieting individuals and those who have never been on a diet [43]; convergent validity: null [43] to strong correlation [48]; construct validity through CFA: χ2/df: adequate fit [42, 51]; CFI: unacceptable fit [51]; RMSEA: good fit; goodness-of-fit index: poor fit [42, 51]; adjusted goodness-of-fit index: poor fit [42]; factor loadings: middle to high level [41]. The EFA results were: Bartlett’s test: statistically significant [42, 43, 46, 49–51]; KMO: average [50] to very good [42, 46]; and Bartlett’s test and KMO not evaluated [25, 44]; factor loadings: minimum [25, 43, 46, 51] to high level [25, 41, 43, 44, 46, 49–51]. The diagnostic performance results were: the AUC was considered high [48] to excellent accuracy [47, 49, 50] and the AUC was not evaluated [44, 45]; internal consistency: Cronbach’s alpha coefficient: unacceptable [41] to excellent [44, 48, 49]; polychoric ordinal alpha: acceptable [43] to excellent [43]. The test–retest results were: Pearson correlation coefficient: null [43] to strong correlation [43, 51]; ICC: good [48] to excellent [46] (see Table 2).

### Children's eating attitudes test (ChEAT)

The ChEAT is a self-report and 26-item instrument that contemplates three domains: dieting attitudes, oral control, and social pressure to restrict eating [14]. The ChEAT-26 was translated into Portuguese and its psychometric properties were evaluated in one study in Brazil. The instrument was validated in a sample of 346 female and male participants, with a mean age of 10 years old. The participant inclusion criteria was pre-adolescents from eight to 12 years old recruited from four private schools.

According to the COSMIN methodological quality assessment, the translation process was considered “doubtful”; the internal consistency assessment was classified as “very good”; and the structural validity examination was considered “adequate” [52]. The content validity, hypotheses testing, criterion validity, test–retest, measurement invariance, and responsiveness were “not reported” (see Fig. 2 and Additional file 3). This study did not contemplate the minimum psychometric assessment (content, criterion, and construct validity) or diagnostic performance.

The psychometric property results according to the described cut-off points were: construct validity: Bartlett’s test: statistically significant [52]; KMO: average [52]; factor loadings: minimum [52] to high level [52]; internal consistency: Cronbach’s alpha coefficient: moderate [52] (see Table 2).

### Overall results

This review included 28 studies conducted in low- and middle-income countries on the psychometric properties of commonly used ED symptom measures. Of those countries, 10 were classified as middle-high income [25–27, 29–33, 35–39, 42, 44–52] and three were classified as lower-middle income [28, 34, 40, 41, 43].

According to the COSMIN assessment, most of the studies were considered as having: a “very good or adequate” (46%) or “doubtful” (10%) translation process; " “very good or adequate” (53%) hypotheses testing; a “very good or adequate” (71%) structural validity examination; and “very good” (96%) internal consistency. Most studies did not report content validity (88%), measurement invariance (89%), criterion validity (71%), or test–retest (57%). No studies reported responsiveness. Thus, according to this classification, most (57%) of the studies did not describe (“not reported”) the psychometric properties assessed (see Fig. 2 and Additional file 3).

Forty-three percent of the studies conducted the minimum psychometric evaluation. According to the described cut-off points, the psychometric properties assessed showed overall acceptable validity and reliability results (see Table 2).

### Performance of original studies that developed and validated the questionnaires according to the COSMIN tool, minimum psychometric evaluation, and cut-off points.

Overall score, (40%) of the studies did not describe (“not reported”) the psychometric properties assessed in this tool [10–12, 14, 62] (see Additional Files 4 and 5). None of the studies conducted the minimum psychometric evaluation.

The following results were obtained according to the cut-off points described: convergent validity: weak [14] to strong [62] correlation; discriminant validity: the questionnaires showed a significant difference between individuals with AN [10, 12], individuals with BN [10], and control groups [10, 12]; construct validity though EFA: Bartlett’s test and KMO not evaluated [12, 14]; factor loadings: minimum [14] to high level [12]; internal consistency: Cronbach’s alpha coefficient: questionable [10] to excellent[12]; and test–retest: Pearson correlation coefficient: strong correlation [14], (see Additional file 6).

### 1.6 Description of individual studies of most validated questionnaires according to the COSMIN tool

Since the EAT and the EDE-Q were the instruments with the most evaluations, they were compared with their original versions to compare the impact of psychometric properties. Forty percent of the original studies did not describe the psychometric properties (Additional files 7 and 8).

### Comparison between studies of validated questionnaires with translation process and those without this process

According to the overall COSMIN score, in both cases, more than 45% of the studies did not describe most of the psychometric properties (see Additional file 9).

## Discussion

Most of the studies conducted in low- and middle-income countries on measures for assessing well-known eating disorder symptoms did not described psychometric properties according to the COSMIN methodological quality classification of the individual studies and they did not conduct the minimum recommended assessment of these properties. However, according to the described cut-off points, the psychometric properties evaluated showed overall acceptable validity and reliability results. In addition, most studies were conducted in middle-income countries.

The overall score for the methodological quality of each measurement according to the COSMIN procedure is determined considering the lowest classification of any one of the items evaluated. While a strength of the COSMIN procedure is that it has very rigid and specific criteria for evaluating psychometric properties, the result is that there is little flexibility in the tool. For example, in the test–retest evaluation, if researchers choose to report Pearson or Spearman correlations instead of the kappa coefficient or ICC, this property could be classified as doubtful [22]. The assessment of psychometric properties involves a vast field and there is still a lack of standardization in conceptual and methodological terms [23]. According to the general COSMIN assessment, most of the original studies also did not describe the psychometric properties evaluated. In addition, most of these studies did not meet the minimum criteria recommended for the evaluation of psychometric properties. These results reinforce the need to properly assess psychometric properties in the development of ED assessment tools.

We also considered the minimum criteria recommended for assessing psychometric properties, and most studies did not include these criteria either. For example, of the 17 studies that carried out the translation process, only two assessed the content validity for the cultural adaptation of the instrument [23]. However, the studies with and without the translation process showed great similarity in the evaluation of the psychometric properties according to the COSMIN score. All the validated tools were developed in English in high-income countries. Understanding the possible cultural difference between countries is crucial for an adequate assessment of the instruments [67, 68].

The psychometric properties were most frequently assessed in the EAT and EDE-Q instruments. A possible explanation for the greater use of these questionnaires may be that both instruments include important domains for the assessment of EDs, and they are already widely used and recognized in clinical practice [69–71]. As with original studies, the majority of the most used questionnaires did not describe the psychometric properties evaluated in COSMIN.

The EDE, one of the most frequently used instruments for measuring EDs, was only validated in China [29, 69]. The ChEDE and ChEDE-Q instruments were not validated in any of the low-and middle-income countries.

Of the 28 studies included in this review, only five received partial funding to develop their research. This emphasizes the scarcity of resources for developing ED research in low- and middle-income countries. A lack of resources can significantly compromise the feasibility of conducting studies according to methodological recommendations [72].

Regarding the limitations of this review, no country-specific databases were accessed. However, the search for evidence included six databases and gray literature and did not include any language or publication date restrictions. Another limitation is that this review included only the most commonly used instruments and did not cover all instruments. In addition, we did not compare the diagnostic performance of questionnaires in studies that evaluated psychometric properties. However, only six studies evaluated the diagnostic performance of three instruments.

## Conclusion

The results of this review suggest a lack of studies in low- and middle-income countries on psychometric properties in commonly used instruments for measuring EDs. With the steady increase in the prevalence of EDs globally, psychometric investigations of instruments for measuring eating disorder symptoms in these countries should be encouraged to promote their early detection and treatment.

## Supplementary Information

**Additional file 1** Database search strategy.

**Additional file 2** Cut-off points for psychometric properties.

**Additional file 3** COSMIN methodological quality classification of individual studies.

**Additional file 4** COSMIN methodological quality classification of original studies.

**Additional file 5** COSMIN methodological quality classification of original studies.

**Additional file 6** Methodology and results of the validation process of the original studies.

**Additional file 7** COSMIN classification of the methodological quality of the EAT from the original study versus the studies included in this review.

**Additional file 8** COSMIN classification of the methodological quality of the EDE-Q from the original study versus the studies included in this review.

**Additional file 9** COSMIN classification of the methodological quality of studies with and without a translation process.

## Data Availability

The datasets analyzed during the current study are available from the corresponding author on reasonable request.

## Abbreviations

AN: Anorexia nervosa
AUC: Area under the curve
BN: Bulimia nervosa
ChEAT: Children's eating attitudes test
ChEDE: Children's eating disorder examination
ChEDE-Q: Children's eating disorder examination questionnaire
CABI: Commonwealth agricultural Bureaux international
CFI: Comparative fit index
CFA: Confirmatory factor analysis
COSMIN: Consensus-based standards for the selection of health measurement instruments
DSM-IV: Diagnostic and statistical manual of mental disorders-IV
DSM-5: Diagnostic and statistical manual of mental disorders-5
DOI: Digital Object Identifier
EAT: Eating attitudes test
EDs: Eating disorders
EDE: Eating disorder examination
EDE-Q: Eating disorder examination-questionnaire
EDE-QS: Eating disorder examination-questionnaire short
EDI: Eating disorder inventory
EDs: Eating disorders
EFA: Exploratory factor analysis
Fig: Figure
ICC: Intraclass correlation coefficients
KMO: Kaiser Meyer Olkin
LILACS: Latin American & Caribbean Health Sciences Literature
PRISMA: Preferred reporting items for systematic reviews and meta-analyses
PROSPERO: International prospective register of systematic reviews
TLI: Tucker Lewis index
SRMSR: Standardized root mean square residual
RMSEA: Root mean square error of approximation
