# Supplementary material for: Assessing eating disorder symptoms in low and middle-income countries: a systematic review of psychometric studies of commonly used instruments
Source: J Eat Disord. 2022 Aug 23;10:124. doi: 10.1186/s40337-022-00649-z (PMC9400307; doi:10.1186/s40337-022-00649-z)
Supplement: Supplementary file 1 — Additional file 1 Database search strategy. [file 40337_2022_649_MOESM1_ESM.docx]

**Additional file 1.** Database search strategy

**MEDLINE (PubMed)**

1. Feeding and Eating Disorders

2. Eating and Feeding Disorders

3. Eating Disorders

4. Disorder, Eating

5. Disorders, Eating

6. Eating Disorder

7. OR/ 1 – 6

8. Questionnaires

9. Questionnaire

10. Self report

11. Self reports

12. OR/ 8 - 11

13. Eating attitudes test

14. EAT

15. Eating attitudes test-40

16. Eating attitudes test 40

17. EAT-40

18. EAT40

19. Eating attitudes test-26

20. Eating attitudes test 26

21. EAT-26

22. EAT26

23. Eating disorder inventory

24. EDI

25. Eating disorder inventory-2

26. Eating disorder inventory 2

27. EDI-2

28. EDI2

29. Eating disorder inventory 3

30. Eating disorder inventory-3

31. EDI3

32. EDI-3

33. Eating disorder examination

34. EDE

35. Eating disorder examination-questionnaire

36. EDE-Q

37. Eating disorder examination questionnaire

38. EDEQ

39. Children's eating attitudes test

40. ChEAT

41. Children's eating disorder examination

42. ChEDE

43. Children's Eating Disorder Examination-Questionnaire

44. ChEDE-Q

45. Children's Eating Disorder Examination Questionnaire

46. ChEDEQ

47. OR/ 16 – 46

48. Psychometrics

49. Psychometric

50. Reproducibility of Results

51. Reliability of Results

52. Test-Retest Reliability

53. Reliabilities, Test-Retest

54. Validation Study

55. Validation Studies

56. Factor Analysis, Statistical

57. Factor Analyses, Statistical

58. Statistical Factor Analyses

59. Statistical Factor Analysis

60. Sensitivity and Specificity

61. Specificity and Sensitivity

62. ROC Curve

63. Curve, ROC

64. Curves, ROC

65. Predictive Value of Tests

66. False Negative Reactions

67. False Negative Reaction

68. Reaction, False Negative

69. Reactions, False Negative

70. False Positive Reactions

71. False Positive Reaction

72. Positive Reaction, False

73. Positive Reactions, False

74. Item Response Theory

75. IRT

76. OR/ 48 – 75

77. 7 AND 12 AND 47 AND 76

**EMBASE**

#1. 'eating disorder'

#2. 'eating disorders'

#3. 'feeding and eating disorder'

#4. 'feeding and eating disorders'

#5. OR/ #1 - #4

#6. 'questionnaires'

#7. 'questionnaire'

#8. 'self report'

#9. 'self reports'

#10. OR/ #6 - #9

#11. 'psychometry'

#12. 'Psychometrics'

#13. 'reproducibility'

#14. 'measurement reproducibility'

#15. 'reproducibility of results'

#16. 'reproductivity'

#17. 'validation study'

#18. 'validation studies'

#19. 'validation studies as topic'

#20. 'factor analysis'

#21. 'analysis, factorial'

#22. 'factor analysis, statistical'

#23. 'factorial analysis'

#24. 'statistical factor analysis'

#25. 'confirmatory factor analysis'

#26. 'confirmatory factorial analysis'

#27. 'exploratory factor analysis'

#28. 'exploratory factorial analysis'

#29. 'sensitivity and specificity'

#30. 'specificity and sensitivity'

#31. 'receiver operating characteristic'

#32. 'receiver operating characteristic curve'

#33. 'ROC curve'

#34. 'predictive value'

#35. 'negative predictive value'

#36. 'positive predictive value'

#37. 'predictive value of tests'

#38. 'Item Response Theory'

#39. 'ITR'

#40. OR/ #11 – #39

#41. #5 AND #10 AND #40

**Web of Science**

#1. TS= (Feeding and Eating Disorders)

#2. TS= (Eating and Feeding Disorders)

#3. TS= (Eating Disorders)

#4. TS= (Disorder, Eating)

#5. TS= (Disorders, Eating)

#6. TS= (Eating Disorder)

#7. OR/ #1 - #6

#8. TS= (Questionnaires)

#9. TS= (Questionnaire)

#10. TS= (Self report)

#11. TS= (Self reports)

#12. OR/ #8 - #11

#13. TS= (Eating attitudes test)

#14. TS= (EAT)

#15. TS= (Eating disorder inventory)

#16. TS= (EDI)

#17. TS= (Eating disorder examination)

#18. TS= (EDE)

#19. TS= (Eating disorder examination-questionnaire)

#20. TS= (EDE-Q)

#21. TS= (Children's eating attitudes test)

#22. TS= (ChEAT)

#23. TS= (Children's eating disorder examination)

#24. TS= (ChEDE)

#25. TS = (Children's Eating Disorder Examination-Questionnaire)

#26. TS= (ChEDE-Q)

#27. OR/ #13 - #26

#28. TS= (Psychometrics)

#29. TS= (Psychometric)

#30. TS= (Reproducibility of Results)

#31. TS= (Reliability of Results)

#32. TS= (Test-Retest Reliability)

#33. TS= (Reliabilities, Test-Retest)

#34. TS= (Validation Study)

#35. TS= (Validation Studies)

#36. TS= (Factor Analysis, Statistical)

#37. TS= (Factor Analyses, Statistical)

#38. TS= (Statistical Factor Analyses)

#39. TS= (Statistical Factor Analysis)

#40. TS= (Sensitivity and Specificity)

#41. TS= (Specificity and Sensitivity)

#42. TS= (ROC Curve)

#43. TS= (Curve, ROC)

#44. TS= (Curves, ROC)

#45. TS= (Predictive Value of Tests)

#46. TS= (False Negative Reactions)

#47. TS= (False Negative Reaction)

#48. TS= (Reaction, False Negative)

#49. TS= (Reactions, False Negative)

#50. TS= (False Positive Reactions)

#51. TS= (False Positive Reaction)

#52. TS= (Positive Reaction, False)

#53. TS= (Positive Reactions, False)

#54. TS= (Item Response Theory)

#55. TS= (IRT)

#56. OR/ #28 - #55

#57. #7 AND #12 AND #27 AND #56

**LILACS**

1. Feeding and Eating Disorders OR Eating and Feeding Disorders OR Eating Disorders OR Disorder, Eating OR Disorders, Eating OR Eating Disorder AND questionnaires OR questionnaire OR self-report OR self-reports [Words]

AND

1. Eating attitudes test OR EAT OR eating attitudes test-40 OR eating attitudes test 40 OR EAT-40 OR EAT40 OR Eating attitudes test-26 OR eating attitudes test 26 OR EAT-26 OR EAT26 OR eating disorder inventory OR EDI OR eating disorder inventory-2 OR eating disorder inventory 2 OR EDI-2 OR EDI2 OR eating disorder inventory 3 OR eating disorder inventory-3 OR EDI3 OR EDI-3 OR eating disorder examination OR EDE OR eating disorder examination-questionnaire OR EDE-Q OR eating disorder examination questionnaire OR EDEQ OR children’s eating attitudes test OR ChEAT OR children’s eating disorder examination OR ChEDE OR Children’s Eating Disorder Examination-Questionnaire OR ChEDE-Q OR Children’s Eating Disorder Examination Questionnaire OR ChEDEQ [Words]

AND

1. Psychometrics OR Psychometric OR Reproducibility of Results OR Reliability of Results OR Test-Retest Reliability OR Reliabilities, Test-Retest OR Validation Study OR Validation Studies OR Factor Analysis, Statistical OR Factor Analyses, Statistical OR Statistical Factor Analyses OR Statistical Factor Analysis OR Sensitivity and Specificity OR Specificity and Sensitivity OR ROC Curve OR Curve, ROC OR Curves, ROC OR Predictive Value of Tests OR False Negative Reactions OR False Negative Reaction OR Reaction, False Negative OR Reactions, False Negative OR False Positive Reactions OR False Positive Reaction OR Positive Reaction, False OR Positive Reactions, False OR Item Response Theory OR IRT [Words]

**PsycInfo (APA PsycNet)**

Any Field: “Feeding and Eating Disorders” OR “Eating and Feeding Disorders” OR “Eating Disorders” OR “Disorder, Eating” OR “Disorders, Eating” OR “Eating Disorder”

AND

Any Field: "questionnaires" OR Any Field: "questionnaire" OR Any Field: "self report" OR Any Field: "self reports"

AND

Any Field: “eating attitudes test” OR “EAT” OR “eating attitudes test-40” OR “eating attitudes test 40” OR “EAT-40” OR “EAT40” OR “Eating attitudes test-26” OR “eating attitudes test 26” OR “EAT-26” OR “EAT26” OR “eating disorder inventory” OR “EDI” OR “eating disorder inventory-2” OR “eating disorder inventory 2” OR “EDI-2” OR “EDI2” OR “eating disorder inventory 3” OR “eating disorder inventory-3” OR “EDI3” OR “EDI-3” OR “eating disorder examination” OR “EDE” OR “eating disorder examination-questionnaire” OR “EDE-Q” OR “eating disorder examination questionnaire” OR “EDEQ” OR “children's eating attitudes test” OR “ChEAT” OR “children's eating disorder examination” OR “ChEDE” OR “Children's Eating Disorder Examination-Questionnaire” OR “ChEDE-Q” OR “Children's Eating Disorder Examination Questionnaire” OR “ChEDEQ”

AND

Any Field: “Psychometrics” OR “Psychometric” OR “Reproducibility of Results” OR “Reliability of Results” OR “Test-Retest Reliability” OR “Reliabilities, Test-Retest” OR “Validation Study” OR “Validation Studies” OR “Factor Analysis, Statistical” OR “Factor Analyses, Statistical” OR “Statistical Factor Analyses” OR “Statistical Factor Analysis” OR “Sensitivity and Specificity” OR “Specificity and Sensitivity” OR “ROC Curve” OR “Curve, ROC” OR “Curves, ROC” OR “Predictive Value of Tests” OR “False Negative Reactions” OR “False Negative Reaction” OR “Reaction, False Negative” OR “Reactions, False Negative” OR “False Positive Reactions” OR “False Positive Reaction” OR “Positive Reaction, False” OR “Positive Reactions, False” OR “Item Response Theory” OR “IRT

**CABI**

"questionnaires"

OR "questionnaire"

OR "self report"

OR "self reports"

AND "eating attitudes test"

OR "EAT"

OR "eating attitudes test-40"

OR "eating attitudes test 40"

OR "EAT-40"

OR "EAT40"

OR "Eating attitudes test-26"

OR "eating attitudes test 26"

OR "EAT-26"

OR "EAT26"

OR "eating disorder inventory"

OR "EDI"

OR "eating disorder inventory-2"

OR "eating disorder inventory 2"

OR "EDI-2"

OR "EDI2"

OR "eating disorder inventory 3"

OR "eating disorder inventory-3"

OR "EDI3"

OR "EDI-3"

OR "eating disorder examination"

OR "EDE"

OR "eating disorder examination-questionnaire"

OR "EDE-Q"

OR "eating disorder examination questionnaire"

OR "EDEQ"

OR "children’s eating attitudes test"

OR "ChEAT"

OR "children's eating disorder examination"

OR "ChEDE"

OR "Children's Eating Disorder Examination-Questionnaire"

OR "ChEDE-Q"

OR "Children's Eating Disorder Examination Questionnaire"

OR "ChEDEQ"

AND "Psychometrics"

OR "Psychometric"

OR "Reproducibility of Results"

OR "Reliability of Results"

OR "Test-Retest Reliability"

OR "Reliabilities, Test-Retest"

OR "Validation Study"

OR "Validation Studies"

OR "Factor Analysis, Statistical"

OR "Factor Analyses, Statistical"

OR "Statistical Factor Analyses"

OR "Statistical Factor Analysis"

OR "Sensitivity and Specificity"

OR "Specificity and Sensitivity"

OR "ROC Curve"

OR "Curve, ROC"

OR "Curves, ROC"

OR "Predictive Value of Tests"

OR "False Negative Reactions"

OR "False Negative Reaction"

OR "Reaction, False Negative"

OR "Reactions, False Negative"

OR "False Positive Reactions"

OR "False Positive Reaction"

OR "Positive Reaction, False"

OR "Positive Reactions, False"

OR "Item Response Theory"

OR "IRT"
