# Supplementary material for: Assessing eating disorder symptoms in low and middle-income countries: a systematic review of psychometric studies of commonly used instruments
Source: J Eat Disord. 2022 Aug 23;10:124. doi: 10.1186/s40337-022-00649-z (PMC9400307; doi:10.1186/s40337-022-00649-z)
Supplement: Supplementary file 2 — Additional file 2 Cut-off points for psychometric properties. [file 40337_2022_649_MOESM2_ESM.docx]

**Additional file 2.** Cut-off points for psychometric properties

| **Psychometric properties** | **Type** | **Method** | **Cut-off points** |
| --- | --- | --- | --- |
| **Content Validity** | Content Validity Index [1,2] | Validity index for clarity | Acceptable standard: > 0.8  Revised or excluded: < 0.8 |
|  |  | Validity index for relevancy |  |
|  |  | Comprehensiveness of the survey |  |
|  |  | Item-total correlations [3] | Poor: ≤0.19  Marginal: 0.2–0.29  Good: 0.3–0.39  Excellent: ≥0.4 |
| **Criterion validity** | Discriminant Validity or  Convergent Validity | Correlation coefficient (r) [4] | Null: 0.10  Weak: 0.10 - 0.39  Moderate: 0.40 - 0.69  Strong: 0.70 - 0.89  Very strong: 0.9 - 1 |
|  |  | Spearman rank correlation coefficients [5] | Poor: <0.3  Fair: 0.3 – 0.5  Moderately strong: 0.5 – 0.8  Very strong: >0.8 |
|  |  | Difference between means [6] | P-Value: statistically significant: ≤0.05 |
| **Construct Validity** | Exploratory Factor Analysis [7,8] | Bartlett's test | Value of P <0.05 |
|  |  | Kaiser-Meyer-Olkin coefficient | Unacceptable: <0.5  Bad: 0.5 - 0.6  Reasonable: 0.6 - 0.7  Average: 0.7 - 0.8  Good: 0.8 - 0.9  Very good: 0.9 -1 |
|  |  | Eigenvalues | Eigenvalue: >1 |
|  |  | Factor Loadings | Minimum level: 0.30  Middle level: 0.40  High level: 0.50 |
|  | Confirmatory Factor Analysis | Relative Chi-Square  Chi-square statistic/degrees of freedom [9,10] | Inadequate fit: >3  Adequate fit (χ2/df): ≤3.00, p<0.05 |
|  |  | Root Mean Square Error of Approximation [8] | Unacceptable adjustment: >0.10 Marginal fit: 0.08 to 0.10  Acceptable fit: 0.05 to 0.08  Good fit: < 0.05 |
|  |  | Comparative Fit Index [11] | Unacceptable adjustment: < 0.90  Good fit: > 0.95 |
|  |  | Taylor-Lewis Index [11] | Unacceptable adjustment: < 0.90  Good fit: >0.95 |
|  |  | Standardized Root Mean Residual [11] | Good fit: <0.08  Acceptable fit: <0.09 |
|  |  | Goodness-of-Fit Index  Adjusted Goodness-of-Fit Index [12] | Poor fit: <0.95  Good fit: >0.95 |
| **Item Response Theory**  **Rasch Modeling**[13,14] | Unidimensionality | Unexplained Variance [15] | Poor: >15%  Fair: 10 – 15%  Good: 5-10%  Very good: 3 - 5%  Excellent: <3% |
|  |  | Variance Explained [15] | Minimum : ≥20%  Moderate: ≥30%  Strong: ≥40% |
|  |  | Eigenvalue [15] | Minimum : <1.5  Acceptable: <3.0  Excellent: <5.0 |
|  |  | Person reliability index [16] | Not acceptable: 0.6  Good: 0.8  Excellent: 0.9 |
|  |  | Person separation index [16] | Acceptable: 1.5  Good: 2.0  Excellent: 3.0 |
|  | Item calibration | Information weighted ft statistic (inft) Outlier-sensitive ft statistic (outft) [17] | Less productive: <0.5  Non-productive: 1.5 to 2.0  Productive: 0.5 to 1.5 |
|  |  | Differential Item Functioning [18] | Negligible: ≥0.43  Intermediate: >0.44 -0.63  Large: ≥0.64 |
| **Diagnostic performance** | Receiver operating characteristic curves (ROC) | Area under the curve[19] | Low accuracy: <0.5  Moderate accuracy: 0.5 - 0.7  High accuracy: 0.7 - 0.9  Excellent: >0.90 |
| **Measurement invariance** [20,21] | Configural and metric invariance | Δ Root Mean Square Error of Approximation | Non invariance: > 0.016  Strong invariance: ≤ 0.015 |
|  |  | Δ Comparative Fit Index | Non invariance: > 0.02  Strong invariance: ≤ 0.01 |
|  |  | Δ Standardized Root Mean Square Residual | Non invariance: > 0.031  Strong invariance: ≤ 0.030 |
| **Reliability** | Internal consistency | Cronbach's alpha coefficients [22] | Unacceptable: <0.50  Poor: >0.50  Questionable: >0.60  Acceptable: > 0.70  Good: > 0.80  Excellent: 0.90 |
|  |  | Ordinal coefficient alpha[23] | Unacceptable: >0.50  Acceptable: >0.60  Good: >0.80  Excellent: 0.90 |
|  |  | Omega coefficients [24] | Poor: <0.50  Questionable: >0.60  Good: >0.80  Excellent: >0.90 |
|  | Test–Retest | Intraclass correlation coefficient [25] | Poor: <0.5  Moderate: 0.5 - 0.75  Good: 0.75 – 0.9  Excellent: >0.9 |
|  |  | Cohen’s Kappa coefficient [26] | Absent: κ <0.10  Poor: κ: 0.11 – 0.40  Discrete: κ: 0.11 – 0.60  Moderate: κ: 0.61 – 0.80  Substantial: 0.81 – 1.0 |
|  |  | Correlation coefficient  (r) [4] | Null: 0  Weak: 0 - 0.3  Regular: 0.3 - 0.6  Strong: 0.6 – 0.9  Very strong: 0.9 - 1  Full or perfect: 1 |

**References**

1. Polit D, Beck C. The Content Validity Index: Are You Sure You Know What’s Being Reported? Critique and Recommendations. Res Nurs Health. 2006;29:488–97. <http://dx.doi.org/10.1002/nur>.

2. Rubio DM, Berg-weger M, Tebb SS, Lee ES, Rauch S. Objectifying content validity : in social work research. 2003;27.

3. Zijlmans EAO, Tijmstra J, van der Ark LA, Sijtsma K. Item-score reliability as a selection tool in test construction. Front Psychol. 2019;9:1–12. <http://dx.doi.org/10.3389/fpsyg.2018.02298>.

4. Schober P, Schwarte LA. Correlation coefficients: Appropriate use and interpretation. Anesth Analg. 2018;126:1763–8. [http://dx.doi.org/10.1213/ANE.0000000000002864.](%20http:/dx.doi.org/10.1213/ANE.0000000000002864.)

5. de Vet HCW, Mokkink LB, Mosmuller DG, Terwee CB. Spearman–Brown prophecy formula and Cronbach’s alpha: different faces of reliability and opportunities for new applications. J Clin Epidemiol. 2017;85:45–9. <http://dx.doi.org/10.1016/j.jclinepi.2017.01.013>.

6. Andrade C. The P Value and Statistical Significance: Misunderstandings, Explanations, Challenges, and Alternatives. Indian J Psychol Med. 2019;41:138–43. http://dx.doi.org/10.4103/IJPSYM.IJPSYM.

7. Watkins MW. Exploratory Factor Analysis: A Guide to Best Practice. J Black Psychol. 2018;44:219–46. http://dx.doi.org/10.1177/0095798418771807.

8. Fabrigar LR, Wegener DT, Maccallum RC, Strahan EJ. Evaluating the Use of Exploratory Factor Analysis in Psychological Research. Psychol Methods. 1999;4:272–99.

9. Fan Y, Chen J, Shirkey G, John R, Wu SR, Park H, et al. Applications of structural equation modeling (SEM) in ecological studies: an updated review. Ecol Process. 2016;5. <http://dx.doi.org/10.1186/s13717-016-0063-3>

10. Curran PJ, Bollen KA, Paxton P, Kirby J, Chen F. The noncentral chi-square distribution in misspecified structural equation models: Finite sample results from a Monte Carlo simulation. Multivariate Behav Res. 2002;37:1–36. <http://dx.doi.org/10.1207/S15327906MBR3701_01>.

11. Hu L, Bentler PM, Hu L. Cutoff criteria for fit indexes in covariance structure analysis : Conventional criteria versus new alternatives Cutoff Criteria for Fit Indexes in Covariance Structure Analysis : Conventional Criteria Versus New Alternatives. 2009;5511. <http://dx.doi.org/10.1080/10705519909540118>.

12. Miles JN, Shevlin M. Effects of sample size, model specification and factor loadings on the GFI in confirmatory factor analysis. Pers Individ Dif. 1998;25:85–90.

13. Chen YL, Pan AW, Chung LI, Chen TJ. Examining the validity and reliability of the Taita symptom checklist using Rasch analysis. J Formos Med Asso. 2015;114:221–30. <http://dx.doi.org/10.1016/j.jfma.2013.10.004>

14. Dabaghi S, Esmaielzadeh F, Rohani C. Application of Rasch Analysis for Development and Psychometric Properties of Adolescents’ Quality of Life Instruments: A Systematic Review. Adolesc Health Med Ther. 2020;11:173–97. <http://dx.doi.org/10.2147/ahmt.s265413.>

15. Linacre J. Data variance explained by measures. Rasch Measurment Transactions; 2006;20:1045– 1047.

16. Linacre J. Winsteps Rasch measurement computer program User’s guide. Beaverton: Winsteps.com; 2012.

17. Linacre J, Wright B. Dichotomous Mean-square Chi-square fit statistics. Rasch Measurement Transactions; 1994;360.

18. Rouquette A, Hardouin JB, Coste J. Differential Item Functioning (DIF) and Subsequent Bias in Group Comparisons using a Composite Measurement Scale: A Simulation Study. J Appl Meas. 2016;17:312–34.

19. Akobeng AK. Understanding diagnostic tests 3: Receiver operating characteristic curves. Acta Paediatr Int J Paediatr. 2007;96:644–7. http://dx.doi.org/[10.1111/j.1651-2227.2006.00178.x](https://doi.org/10.1111/j.1651-2227.2006.00178.x).

20. Cheung GW, Rensvold RB. Evaluating goodness-of-fit indexes for testing measurement invariance. Struct Equ Model. 2002;9:233–55. <http://dx.doi.org/10.1207/S15328007SEM0902_5>.

21. Chen FF. Sensitivity of goodness of fit indexes to lack of measurement invariance. Struct Equ Model. 2007;14:464–504. [http://dx.doi.org/10.1080/10705510701301834.](%20http:/dx.doi.org/10.1080/10705510701301834.)

22. Taber KS. The Use of Cronbach ’ s Alpha When Developing and Reporting Research Instruments in Science Education. Research in Science Education. 2018;1273–96. <http://dx.doi.org/10.1007/s11165-016-9602-2>.

23. Zumbo BD, Gadermann AM, Zeisser C. Ordinal versions of coefficients alpha and theta for likert rating scales. J Mod Appl Stat Methods. 2007;6:21–9. <http://dx.doi.org/10.22237/jmasm/1177992180.>

24. Viladrich C, Angulo-Brunet A, Doval E. A journey around alpha and omega to estimate internal consistency reliability. An psicol. 2017;33:755–82. 1 <http://dx.doi.org/0.6018/analesps.33.3.268401>.

25. Koo TK, Li MY. A Guideline of Selecting and Reporting Intraclass Correlation Coefficients for Reliability Research. J Chiropr Med. 2016;15:155–63. <http://dx.doi.org/10.1016/j.jcm.2016.02.012>

26. Shrout PE. Measurement reliability and agreement in psychiatry. Stat Methods Med Res. 1998;7:301–17. http://dx.doi.org/[10.1177/096228029800700306](https://doi.org/10.1177/096228029800700306)
