# Supplementary material for: Assessing eating disorder symptoms in low and middle-income countries: a systematic review of psychometric studies of commonly used instruments
Source: J Eat Disord. 2022 Aug 23;10:124. doi: 10.1186/s40337-022-00649-z (PMC9400307; doi:10.1186/s40337-022-00649-z)
Supplement: Supplementary file 3 — Additional file 3 COSMIN methodological quality classification of individual studies. [file 40337_2022_649_MOESM3_ESM.docx]

**Additional file 3.** COSMIN methodological quality of individual studies classification

| First Author,  Publication year | Instrument Validation | Translation process | Content validity | Hypotheses testing | Structural validity | Criterion validity | Internal consistency | Reliability  Test-retest | Measurement invariance | Responsiveness |
| --- | --- | --- | --- | --- | --- | --- | --- | --- | --- | --- |
| Tong J, 2011 | Eating Disorder Examination | Doubtful | NR | Adequate | NR | Adequate | Very good | Doubtful | NR | NR |
| Penelo E, 2013 | Eating Disorder  Examination- Questionnaire | NA | NA | Very good | Very good | NR | Very good | Very good | Very good | NR |
| Becker AE, 2010 | Eating Disorder  Examination- Questionnaire | Adequate | NR | Adequate | Adequate | NR | Very good | Very good | NR | NR |
| Mahmoodi M, 2016 | Eating Disorder  Examination- Questionnaire | Very good | NR | Adequate | NR | NR | Very good | NR | NR | NR |
| Lewis-Smith H, 2020 | Eating Disorder  Examination- Questionnaire | Adequate | NR | Very good | Very good | NR | Very good | Very good | Inadequate | NR |
| Unikel-Santoncini C, 2018 | Eating Disorder  Examination- Questionnaire | Very good | NR | NR | Very good | NR | Very good | NR | NR | NR |
| He JB, 2021 | Eating Disorder  Examination- Questionnaire-Short | Very good | NR | Adequate | Very good | NR | Very good | Very good | NR | NR |
| Yucel B, 2011 | Eating Disorder  Examination- Questionnaire | Very good | NR | Adequate | NR | NR | Very good | Doubtful | NR | NR |
| Ramli M, 2008 | Eating Disorder  Examination- Questionnaire | Adequate | NR | NR | Very good | NR | Very good | NR | NR | NR |
|  |  |  |  |  |  |  |  |  |  |  |
| Mohd Taib N, 2021 | Eating Disorder  Examination- Questionnaire | Adequate | NR | Adequate | Adequate | NR | Very good | Doubtful | NR | NR |
| Compte EJ, 2019 | Eating Disorder  Examination- Questionnaire | NA | NA | NR | Very good | NR | Very good | NR | Very good | NR |
| Unikel-Santoncini C, 2006 | Eating Disorder Inventory | NA | NA | Adequate | Adequate | NR | Very good | NR | NR | NR |
| García-García E, 2003 | Eating Disorder Inventory - 2 | Inadequate | NR | NR | NR | Very good | Very good | NR | NR | NR |
| Rutsztein G, 2013 | Eating Disorder Inventory - 3 | NA | NA | NR | Adequate | NR | Very good | NR | NR | NR |
| Dadgostar H, 2017 | Eating Disorder Inventory - 3 | Adequate | Very good | NR | NR | NR | Very good | Very good | NR | NR |
| Savaşır I, 1989 | Eating Attitudes Test - 40 | Doubtful | NR | NR | Adequate | NR | Very good | NR | NR | NR |
| Nasser M, 1994 | Eating Attitudes Test - 40 | NA | NA | NR | Adequate | NR | Very good | NR | NR | NR |
| Alvarez-Rayón G, 2004 | Eating Attitudes Test - 40 | NA | NA | Very good | Adequate | Inadequate | Very good | NR | NR | NR |
| Nunes MA, 2005 | Eating Attitudes Test - 26 | NA | NA | NR | NR | Very good | Very good | NR | NR | NR |
| Fortes LS, 2016 | Eating Attitudes Test - 26 | NA | NA | Adequate | Adequate | NR | Very good | Adequate | NR | NR |
| Kang Q, 2017  Constaín GA, 2014 | Eating Attitudes Test - 26  Eating Attitudes Test - 26 | NA  NA | NA  NA | Very good  NR | NR  Adequate | Very good  Very good | Very good  Very good | Very good  NR | NR  NR | NR  NR |
| Constaín GA, 2017 | Eating Attitudes Test - 26 | NA | NA | NR | Adequate | Very good | Very good | NR | NR | NR |
| Erguney-Okumus FE, 2020 | Eating Attitudes Test - 26 | Adequate | NR | Very good | Very good | NR | Very good | Doubtful | NR | NR |
| Kaewporndawan T, 2013 | Eating Attitudes Test - 26 | Very good | Very good | Very good | NR | Very good | NR | NR | NR | NR |
| Haddad C, 2020 | Eating Attitudes Test - 26 | Very good | NR | NR | Very good | NR | Very good | NR | NR | NR |
| Ahmadi S, 2014 | Eating Attitudes Test - 26 | Very good | NR | Very good | Adequate | NR | Very good | Doubtful | NR | NR |
| Pinheiro N, 2012 | Children's Attitudes Test | Doubtful | NR | NR | Adequate | NR | Very good | NR | NR | NR |

*NR not reported; NA: not applicable. Note: Hypotheses testing represents: convergent and discriminant validity; structural validity considers: construct validity and criterion validity represents: diagnostic process.*
