# Supplementary material for: Assessing eating disorder symptoms in low and middle-income countries: a systematic review of psychometric studies of commonly used instruments
Source: J Eat Disord. 2022 Aug 23;10:124. doi: 10.1186/s40337-022-00649-z (PMC9400307; doi:10.1186/s40337-022-00649-z)
Supplement: Supplementary file 4 — Additional file 4 COSMIN methodological quality classification of original studies. [file 40337_2022_649_MOESM4_ESM.docx]

**Additional file 4.** COSMIN methodological quality of original studies classification

| First Author,  Publication year | Instrument Validation | Translation process | Content validity | Hypotheses testing | Structural validity | Criterion validity | Internal consistency | Reliability  Test-retest | Measurement invariance | | Responsiveness | |  |  |  |  |
| --- | --- | --- | --- | --- | --- | --- | --- | --- | --- | --- | --- | --- | --- | --- | --- | --- |
| Garner, D, 1982 | Eating Attitude Test | NA | NR | Adequate | NR | NR | Very good | NR | NR | | NR | |  |  |  |  |
| Garner, D, 1983 | Eating Disorder  Inventory | NA | NR | Very good | Very good | NR | Very good | NR | NR | | NR | |  |  |  |  |
| Cooper, Z, 1987 | Eating Disorder Examination | NA | NR | Adequate | NR | NR | Very good | NR | NR | | NR | |  |  |  |  |
| Maloney, M, 1988 | Children Eating Attitudes Test | NA | NR | Adequate | Adequate | NR | Very good | Very good | NR | | NR | |  |  |  |  |
| Fairburn, C, 1994 | Eating Disorder Examination Questionnaire | NA | NR | Adequate | NR | NR | Very good | NR | NR | | NR | | |  |  |  |
|  |  |  |  |  |  |  |  |  |  |  | |  | | |  |  |

*NR not reported; NA: not applicable. Note: hypotheses testing represents convergent and discriminant validity; structural validity considers: construct validity and criterion validity represents: diagnostic process.*
