# Supplementary material for: Assessing eating disorder symptoms in low and middle-income countries: a systematic review of psychometric studies of commonly used instruments
Source: J Eat Disord. 2022 Aug 23;10:124. doi: 10.1186/s40337-022-00649-z (PMC9400307; doi:10.1186/s40337-022-00649-z)
Supplement: Supplementary file 5 — Additional file 5 COSMIN methodological quality classification of original studies. [file 40337_2022_649_MOESM5_ESM.pptx]

## Slide 1
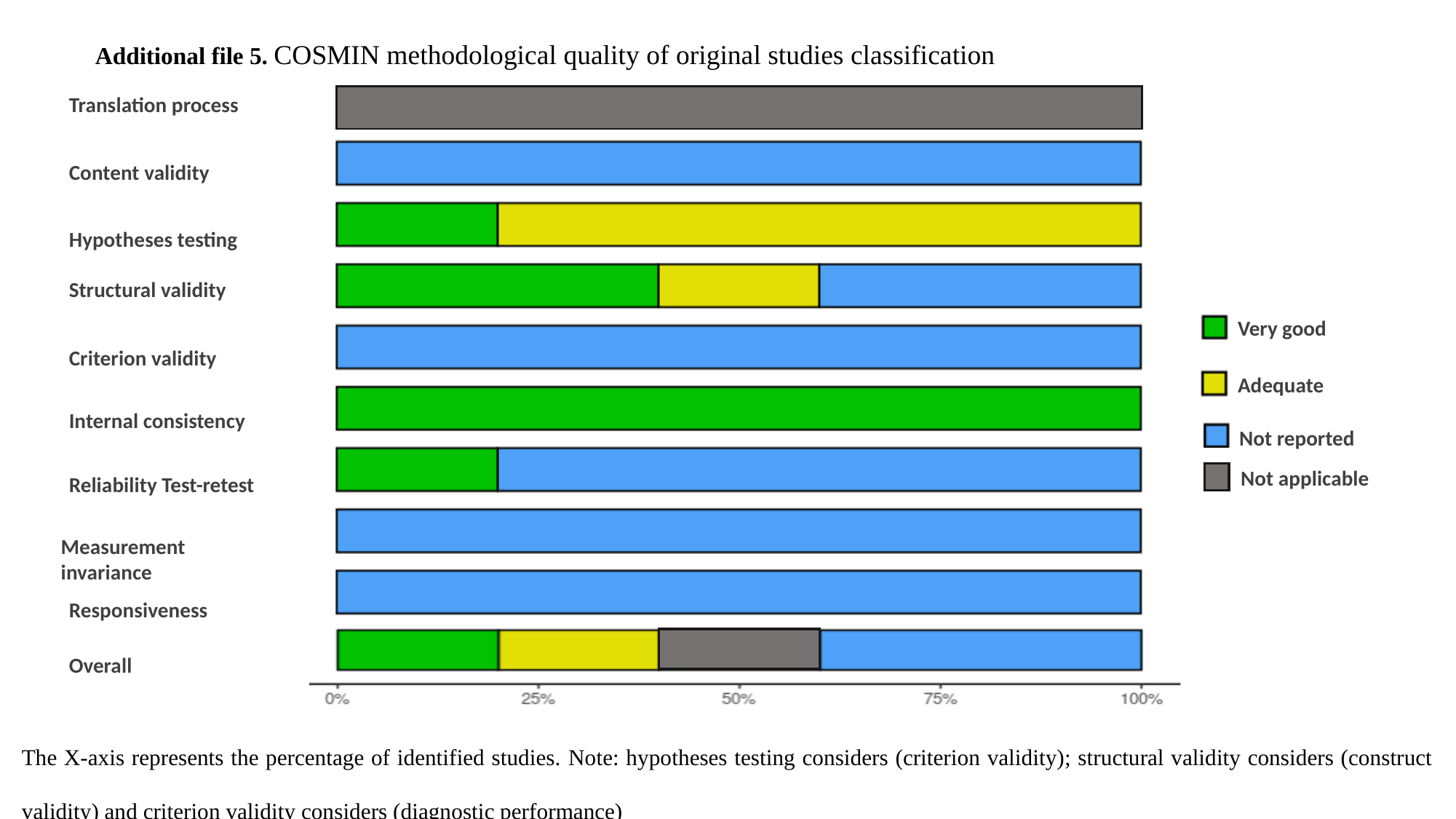

Additional file 5. COSMIN methodological quality of original studies classification
Translation process
Content validity
Hypotheses testing
Structural validity
Criterion validity
Internal consistency
Reliability Test-retest
Measurement invariance
Responsiveness
Overall
Very good
Adequate
Not reported
Not applicable
The X-axis represents the percentage of identified studies. Note: hypotheses testing considers (criterion validity); structural validity considers (construct validity) and criterion validity considers (diagnostic performance)
