# Supplementary material for: Assessing eating disorder symptoms in low and middle-income countries: a systematic review of psychometric studies of commonly used instruments
Source: J Eat Disord. 2022 Aug 23;10:124. doi: 10.1186/s40337-022-00649-z (PMC9400307; doi:10.1186/s40337-022-00649-z)
Supplement: Supplementary file 6 — Additional file 6 Methodology and results of the validation process of the original studies. [file 40337_2022_649_MOESM6_ESM.docx]

**Additional file 6.** Methodology and results of the validation process of the original studies

| First author (Publication year) | Validity methodology | Reliability | Validation results | Reliability results |
| --- | --- | --- | --- | --- |
| Garner, D  (1982) | **Convergent validity**  Eating Attitudes test - 26 **Method**:  Pearson correlation coefficient    **Discriminant validity**  Individuals with anorexia nervosa  Control group  Method:  **Construct validity**  **Exploratory factor analysis**  Method:  Rotation: oblique  Variance total  Factor loadings | **Internal consistency**  Method:  Cronbach’s alpha coefficient | **Convergent validity**  Eating Attitudes Test – 26: r: 0.98; p<0.001 | **Internal consistency**  Cronbach’s alpha coefficient: 0.80 |
| Garner, D (1983) |  | **Internal consistency**  Method:  Cronbach’s alpha coefficient | **Discriminant validity**  Individuals with anorexia nervosa: mean: 52.9, SD: 23.0  Control group: mean: 15.4, SD: 11.0  p< 00001  **Construct validity**  **Exploratory factor analysis**  Eating attitudes test - 40  Factor loadings: all items were >0.40 with range 0.41 to 0.81 | **Internal consistency** Cronbach's alpha coefficient: range: 0.83 to 0.92 |
| Cooper Z, (1987) | **Discriminant validity**  Individuals with anorexia nervosa  Individuals with bulimia nervosa  Control groups  Method:  Mann-Whitney U test | **Internal consistency**  Method:  Cronbach’s alpha coefficient | **Discriminant validity**  Individuals with anorexia nervosa: mean: 2.4; SD: 1.4  Individuals with bulimia nervosa: mean: 3.1; SD: 1.22  Control groups: mean: 0.54; SD: 0.69  P<0.001 | **Internal consistency**  Cronbach’s alpha coefficient: range: 0.67 to 0.79 |
| Maloney (1988) | **Convergent validity**  Weight management behavior  Body dissatisfaction  Method:  Pearson correlation coefficient  **Construct validity**  **Exploratory factor analysis**  Method:  Principal components analysis  Rotation: Varimax  Eigenvalues  Variance total  Factor loadings | **Internal consistency:** Method:  Cronbach's alpha coefficient  **Test-retest:**  Pearson correlation coefficient | **Convergent validity**  Weight management behavior: r: 0.36  Body dissatisfaction: r: 0.39  p < 0.001  **Construct validity**  **Exploratory factor analysis**  Eigenvalues: >1.0 for seven factors  Variance total: 61% for seven factors  Factor loadings: all items had factor loadings >0.30, range: 0.32 to 0.83 | **Internal consistency** Cronbach's alpha coefficient: 0.76  **Test-retest:**  Pearson correlation coefficient: 0.81 |
| Fairburn, C (1994) | **Convergent validity**  Eating Disorder Examination  Method:  Pearson coefficient | **Internal consistency**  Method:  Cronbach’s alpha coefficient  **Test-retest**  Method:  Pearson correlation coefficient | **Convergent validity**  Eating Disorder Examination: r: 0.84; P<0.001 | **Internal consistency**  Cronbach’s alpha coefficient: range: 0.81 to 0.92  **Test-retest**  Pearson correlation coefficient: range: 0.54 to 0.94 |
|  |  |  |  |  |

*SD Standard deviation*
