# Supplementary material for: Assessing eating disorder symptoms in low and middle-income countries: a systematic review of psychometric studies of commonly used instruments
Source: J Eat Disord. 2022 Aug 23;10:124. doi: 10.1186/s40337-022-00649-z (PMC9400307; doi:10.1186/s40337-022-00649-z)
Supplement: Supplementary file 7 — Additional file 7 COSMIN classification of the methodological quality of the EAT from the original study versus the studies included in this review. [file 40337_2022_649_MOESM7_ESM.pptx]

## Slide 1
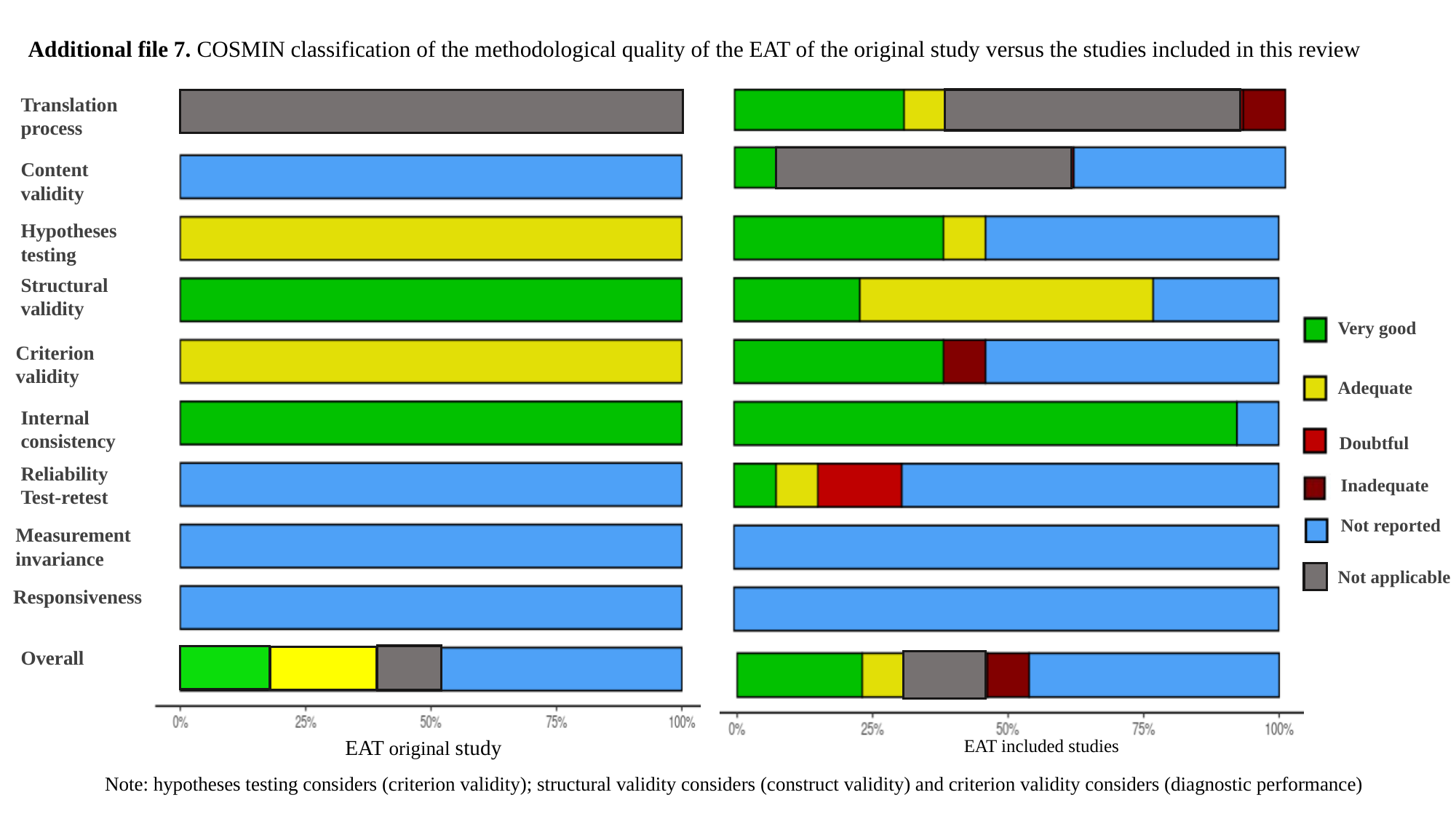

Additional file 7. COSMIN classification of the methodological quality of the EAT of the original study versus the studies included in this review
Translation process
Content validity
Hypotheses testing
Structural validity
Criterion validity
Internal consistency
Reliability Test-retest
Measurement invariance
Responsiveness
Overall
Very good
Adequate
Doubtful
Inadequate
Not reported
Not applicable
EAT original study
EAT included studies
Note: hypotheses testing considers (criterion validity); structural validity considers (construct validity) and criterion validity considers (diagnostic performance)
