# Supplementary material for: Assessing eating disorder symptoms in low and middle-income countries: a systematic review of psychometric studies of commonly used instruments
Source: J Eat Disord. 2022 Aug 23;10:124. doi: 10.1186/s40337-022-00649-z (PMC9400307; doi:10.1186/s40337-022-00649-z)
Supplement: Supplementary file 8 — Additional file 8 COSMIN classification of the methodological quality of the EDE-Q from the original study versus the studies included in this review. [file 40337_2022_649_MOESM8_ESM.pptx]

## Slide 1
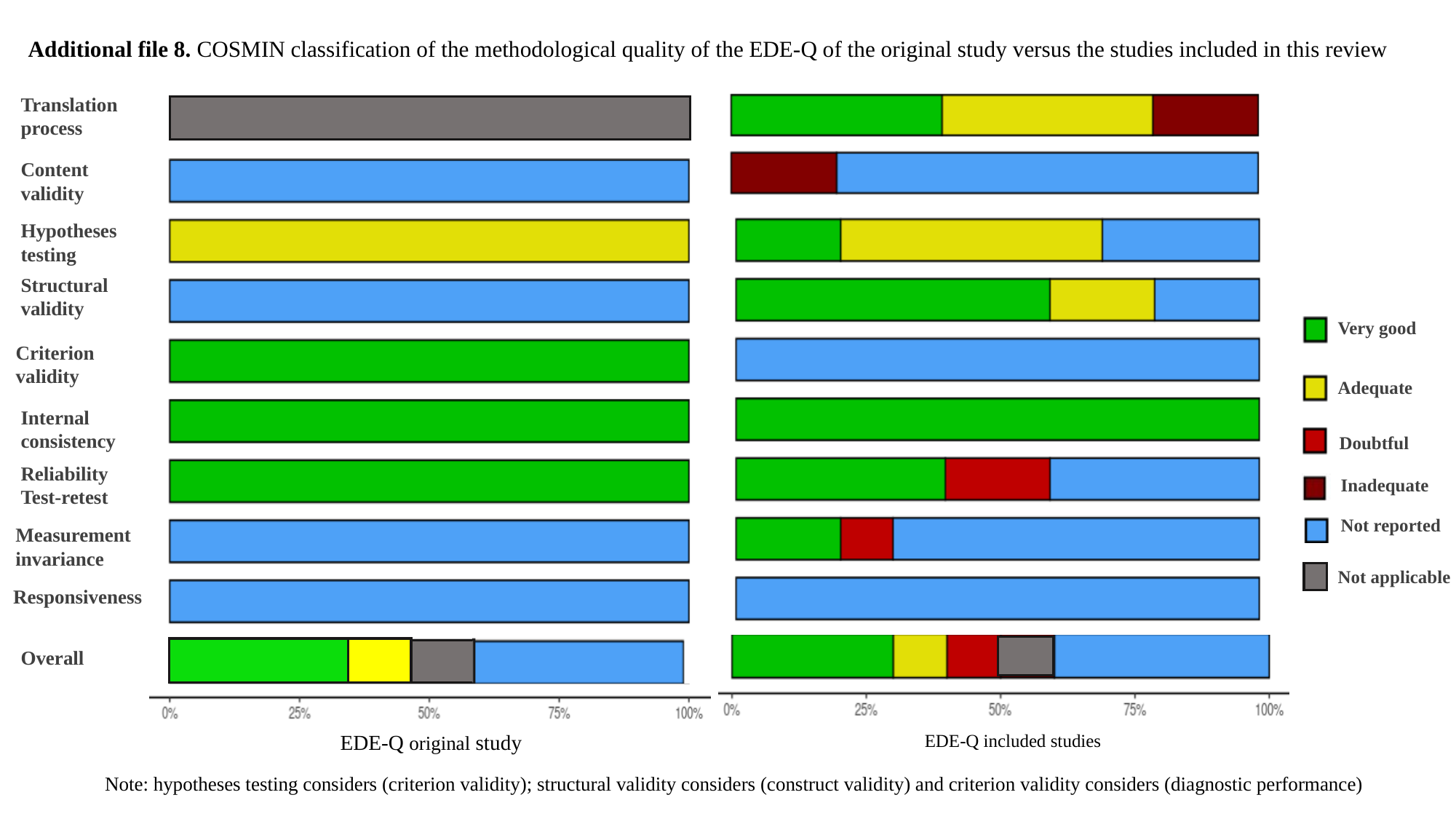

Additional file 8. COSMIN classification of the methodological quality of the EDE-Q of the original study versus the studies included in this review
Translation process
Content validity
Hypotheses testing
Structural validity
Criterion validity
Internal consistency
Reliability Test-retest
Measurement invariance
Responsiveness
Overall
Very good
Adequate
Doubtful
Inadequate
Not reported
Not applicable
EDE-Q included studies
EDE-Q original study
Note: hypotheses testing considers (criterion validity); structural validity considers (construct validity) and criterion validity considers (diagnostic performance)
